# Extracellular vesicle-delivered siRNA targeting RCN1 suppresses acute myeloid leukemia through TFAM-dependent mtDNA-cGAS-STING signaling

**DOI:** 10.20517/evcna.2026.36

**Published:** 2026-07-31

**Authors:** Huan Chen, Na An, Linlin Yang, Jin Lou, Yuming Pan, Minh T.N. Le, Xin Du, Qiaoxia Zhang

**Affiliations:** ^1^Shenzhen Bone Marrow Transplantation Public Service Platform, Shenzhen Institute of Hematology, Shenzhen Second People’s Hospital, First Affiliated Hospital of Shenzhen University, Shenzhen University Health Sciences Center, Shenzhen 518025, Guangdong, China.; ^2^Institute for Digital Medicine and Department of Pharmacology, Yong Loo Lin School of Medicine, National University of Singapore, Singapore 117600, Singapore.

**Keywords:** Extracellular vesicles, reticulocalbin-1, acute myeloid leukemia, mitochondrial DNA, cGAS-STING pathway, siRNA therapeutics

## Abstract

**Aim:** Acute myeloid leukemia (AML) remains a highly aggressive hematologic malignancy with limited therapeutic options. This study aimed to elucidate how reticulocalbin-1 (RCN1) regulates mitochondrial DNA (mtDNA)-mediated innate immune signaling in AML and to evaluate the therapeutic potential of extracellular vesicle (EV)-mediated small interfering RNA (siRNA) delivery targeting RCN1.

**Methods:** Stable RCN1 knockdown was achieved in AML cells using lentiviral delivery of short hairpin RNA. Mitochondrial transcription factor A (TFAM) expression, mtDNA leakage, and activation of the cyclic GMP-AMP synthase (cGAS)-stimulator of interferon genes (STING)-type I interferon pathway were analyzed by immunoblotting and quantitative polymerase chain reaction. Protein interactions were assessed by co-immunoprecipitation, proximity ligation assay, and immunofluorescence. TFAM rescue experiments were performed to evaluate its contribution to the downstream effects of RCN1 knockdown. For translational evaluation, EVs were isolated and characterized by transmission electron microscopy, nanoparticle tracking analysis, and marker protein detection, followed by loading with siRNA targeting RCN1. Therapeutic efficacy and safety were assessed in an AML xenograft mouse model.

**Results:** RCN1 knockdown reduced TFAM expression, leading to cytoplasmic mtDNA accumulation and activation of the cGAS-STING-type I interferon signaling pathway. Restoration of TFAM expression attenuated mtDNA leakage and downstream signaling. EV-delivered siRCN1 effectively suppressed tumor growth *in vivo* without detectable toxicity.

**Conclusion:** RCN1 silencing destabilizes mtDNA integrity and activates innate immune signaling in AML. EV-based delivery of siRCN1 may represent a promising and potentially safe therapeutic strategy for AML.

## INTRODUCTION

Acute myeloid leukemia (AML) is an aggressive hematologic malignancy characterized by the uncontrolled expansion of poorly differentiated myeloid progenitors that accumulate in the bone marrow and peripheral circulation, ultimately impairing normal hematopoiesis and creating a high risk of recurrence and poor clinical outcomes^[1-3]^. Conventional therapeutic options include hypomethylating agents, cytotoxic chemotherapy, and hematopoietic stem cell transplantation. However, clinical outcomes remain unsatisfactory^[3,4]^. Advances in targeted therapies, particularly those directed against B-cell lymphoma 2, FMS-like tyrosine kinase 3, and isocitrate dehydrogenase, have substantially enhanced therapeutic outcomes^[5]^. Nevertheless, relapse remains a major clinical challenge, occurring in 40%-50% of younger adults and at even higher rates in elderly patients, and is associated with limited therapeutic options and poor survival^[4,6]^. Accordingly, there is a critical need to identify new therapeutic targets for AML.

Reticulocalbin-1 (RCN1) is a Ca^2+^-binding protein frequently overexpressed in several malignancies. Studies in solid tumors, including lung cancer and prostate cancer, have demonstrated that elevated RCN1 expression is associated with tumor progression, invasion, poor prognosis, and therapeutic resistance, supporting its broader oncogenic role across multiple malignancies^[7-10]^. Accumulating evidence indicates that RCN1 is a promising therapeutic target for AML^[11]^. The downregulation of RCN1 inhibits cell proliferation and induces pyroptosis via type I interferon signaling, with evidence indicating involvement of the cyclic GMP-AMP synthase (cGAS)-stimulator of interferon genes (STING) pathway based on STING inhibitor (STING-I) assays^[11]^. Cytosolic cGAS functions as a key innate immune sensor that recognizes double-stranded DNA (dsDNA), including mtDNA^[12]^. In solid tumors, chromosomal instability and cytosolic DNA accumulation often trigger cGAS-STING signaling, acting as a double-edged sword, modulating microenvironmental anti-tumor immunity and tumor progression^[13,14]^. In contrast, cGAS-STING activation in AML has been more closely linked to mitochondrial dysfunction and mtDNA leakage, leading to intrinsic inflammatory signaling and cell death programs^[11,15]^. However, the molecular mechanisms connecting RCN1 to mitochondrial homeostasis and cGAS-STING activation in AML remain largely unknown. Therefore, elucidating how RCN1 regulates mtDNA-mediated cGAS-STING signaling may provide mechanistic insights into AML pathogenesis and identify novel therapeutic opportunities.

The mechanisms underlying mtDNA leakage are multifaceted. In addition to the well-studied pores (e.g., Bax/Bak pores, VDAC1 oligomer pores, and mitochondrial permeability transition pores)^[16]^, functional impairment of core mitochondrial proteins, such as mitochondrial transcription factor A (TFAM), represents another important mechanism^[17,18]^. TFAM plays a critical role in maintaining mtDNA stability and regulating its copy number. TFAM deficiency compromises mtDNA integrity and promotes cytosolic accumulation of mtDNA, thereby triggering cGAS-STING signaling^[19,20]^. Therapeutic manipulation of mtDNA-mediated innate immune activation may therefore provide a novel strategy for tumor intervention via the cGAS-STING axis^[21,22]^.

Although RCN1 downregulation shows therapeutic promise, the development of a safe and clinically translatable gene-silencing strategy remains a critical challenge. Small interfering RNA (siRNA) therapeutics offer sequence-specific gene silencing with relatively rapid development and flexible design^[23,24]^. However, naked siRNA is inherently unstable and rapidly degraded *in vivo*, necessitating an efficient and safe delivery platform^[25]^. While viral and synthetic delivery systems such as cationic polymers, liposomes, and cell-penetrating peptides (CPPs) have been developed, they are often limited by immunogenicity, safety concerns, or off-target toxicity^[26-28]^. In recent years, extracellular vesicles (EVs) have emerged as promising delivery vehicles owing to their favorable biocompatibility, low immunogenicity, and intrinsic capacity for nucleic acid transport^[29]^. Among the various EV sources, red blood cell-derived extracellular vesicles (RBCEVs) possess several unique advantages, including abundant availability, the absence of nuclear and mitochondrial DNA (mtDNA), favorable safety profiles, and efficient RNA-loading capacity^[30,31]^. Importantly, previous studies have demonstrated that RBCEVs preferentially accumulate in the liver, spleen, and bone marrow, which coincide with the major anatomical sites of AML involvement^[32]^. This spatial concordance between the natural biodistribution of RBCEVs and the tissue tropism of AML provides a disease-matched delivery strategy that may enhance therapeutic efficiency.

In this study, we investigated whether RCN1 regulates mitochondrial homeostasis and mtDNA-mediated innate immune signaling in AML. We identified an interaction between RCN1 and TFAM mediated by the scaffold protein ATPase family AAA-domain-containing 3A (ATAD3A). RCN1 downregulation reduces TFAM levels, leading to mtDNA leakage and the cGAS-STING-type I interferon response. To bridge mechanistic discovery with translational application, we further employed EV-mediated delivery of siRNA targeting RCN1, which significantly suppressed tumor progression in AML xenograft models without observable toxicity. These findings reveal a novel molecular mechanism and support EV-based RCN1 silencing as a potentially safe and translatable therapeutic strategy for AML.

## METHODS

### Cell culture and infection

The human AML cell lines NB4 (RRID: CVCL_0005), THP-1 (RRID: CVCL_0006), and OCI-AML3 (RRID: CVCL_1844) were sourced from the National Collection of Authenticated Cell Cultures (Shanghai, China) and cultured in RPMI-1640 medium (Gibco, USA) containing 10% heat-inactivated fetal bovine serum (FBS) (Corning, USA) and 1% penicillin-streptomycin (Gibco, USA). THP-1 cells used in this study were maintained in their undifferentiated state without phorbol 12-myristate 13-acetate (PMA) treatment and were selected as a representative AML cell model rather than a macrophage-like uptake model. HeLa cells (RRID: CVCL_0030) were obtained from Shanghai Genechem Co., Ltd. (Shanghai, China) and maintained in DMEM (Gibco, USA) supplemented with 10% heat-inactivated FBS (Corning, USA) and 1% penicillin-streptomycin (Gibco, USA). All cells were cultured at 37 °C in a 5% CO_2_ incubator. The identities of all cell lines were verified by short tandem repeat analysis conducted within the past three years. NB4 cells were authenticated by VivaCell Biotechnology Co., Ltd. on July 17, 2023; THP-1 cells were authenticated by VivaCell Biotechnology Co., Ltd. on May 5, 2023; OCI-AML3 cells were authenticated by Procell Life Science & Technology Co., Ltd. on February 14, 2025; and HeLa cells were authenticated by the Cell Bank of the Chinese Academy of Sciences on April 9, 2025. Cells between passages 5 and 20 were used for all experiments. For infection, cells were treated with the following viruses provided by Shanghai Genechem Co., Ltd. (Shanghai, China): short hairpin negative control lentivirus (shNC), RCN1 knockdown lentivirus (shRCN1), TFAM knockdown lentivirus (shTFAM), RCN1 overexpression lentivirus, and TFAM overexpression lentivirus. The targeting sequences used for the lentiviral transduction are listed in Supplementary Table 1. Following infection, stably transduced cell populations were obtained through puromycin selection (1-2 μg·mL^-1^; InvivoGen, USA). Throughout the study, all cells tested negative for mycoplasma contamination.

### RNA extraction

RNA was extracted using a Steady Pure Universal RNA Extraction Kit II (AG21022, Accurate Biology, China) according to the manufacturer’s instructions. A NanoDrop One (Thermo Fisher Scientific, USA) was used to determine RNA quality and concentration. Subsequently, 1 μg of total RNA was reverse-transcribed into cDNA using an Evo M-MLV Plus 1st Strand cDNA Synthesis Kit (AG11615; Accurate Biology, China). Quantitative polymerase chain reaction (qPCR) was performed using a SYBR Green Pro Taq HS Premix qPCR Kit III (AG11738; Accurate Biology, China) and target-specific primers, with glyceraldehyde-3-phosphate dehydrogenase (*GAPDH*) as the normalization control. The corresponding primer sequences are shown in Supplementary Table 2. All qPCR assays were performed at least three times on a 7300plus Real-Time PCR System (Applied Biosystems, USA).

### Extraction and detection of cytosolic mtDNA

Cells in the control and treated groups were equally divided into two fractions. The first fraction was exposed to 500 μL of 50 mM NaOH and heated for 30 min to ensure complete solubilization of cellular DNA. Following incubation, the alkaline solution was neutralized with 50 μL of 1 M Tris-HCl (pH 8.0). The resulting lysate was used as a normalization control for total mtDNA content, whereas the remaining fraction was resuspended in 500 µL of extraction buffer composed of 150 mM NaCl, 50 mM HEPES (pH 7.4), and 20 μg·mL^-1^ digitonin, followed by gentle rotation at room temperature for 15 min to induce partial plasma membrane permeabilization. Samples were centrifuged at 17,000 × *g* for 10 min, and the cytosolic fraction was obtained from the supernatant. DNA was extracted from the cytosolic fraction using a QIAamp DNA Mini Kit (51304; Qiagen, Germany) following the manufacturer’s instructions. qPCR was performed on whole-cell lysates and cytosolic fractions with primers targeting nuclear DNA [nDNA; β2-microglobulin (*β2M*)] and mtDNA [cytochrome c oxidase subunit I (*COXI*)]. The mtDNA abundance in each fraction was quantified using cycle threshold (CT) values. A complete list of primer sequences is provided in Supplementary Table 2.

### Western blot analysis

To prepare the protein samples, cells were incubated on ice for 30 min in radioimmunoprecipitation assay (RIPA) buffer (Thermo Fisher Scientific, USA) containing protease inhibitors (HY-K0010; MedChemExpress), while RBCEVs were lysed for 15 min. The resulting supernatants were denatured by boiling, and equal amounts of total protein (20 μg per lane) were loaded onto 4%-20% MeilunGel Precast Page Gel (MA04161-3; MeilunBio, China) alongside a PageRuler^TM^ Prestained Protein Ladder (26617; Thermo Fisher Scientific). Following electrophoresis, proteins were blotted onto polyvinylidene fluoride membranes using a standard wet-transfer apparatus. The membranes were blocked with 5% skim milk for 2 h at room temperature and washed twice with TBST (50 mM Tris, pH 8.0, 150 mM NaCl, and 0.1% Tween-20). The membranes were then incubated overnight at 4 °C with the following designated primary antibodies: anti-RCN1 (1:1,000, ab198996; Abcam), anti-β-actin (1:1,000, 4970; Cell Signaling Technology), anti-cGAS (1:1,000, 15102; Cell Signaling Technology), anti-STING (1:1,000, 13647; Cell Signaling Technology), anti-phospho-STING (1:200, 50907; Cell Signaling Technology), anti-TBK1 (1:1,000, 3504; Cell Signaling Technology), anti-phospho-TBK1 (1:1,000, 5483; Cell Signaling Technology), anti-OAS3 (1:1,000, 21915; Proteintech), anti-TFAM (1:1,000, ab272885; Abcam), and anti-ATAD3A (1:1,000, H00055210-D01P; Novus Biologicals). Following an additional TBST wash, the membranes were incubated with horseradish peroxidase-conjugated secondary antibodies diluted 1:5000 for 1 h at room temperature: anti-mouse (7076; Cell Signaling Technology), anti-rabbit (7074; Cell Signaling Technology), and mouse anti-rabbit immunoglobulin G (IgG) conformation-specific monoclonal antibody (5127; Cell Signaling Technology). Protein signals were detected using Immobilon® ECL HRP Substrate (WBULS0100; Millipore) and imaged with a Bio-Rad ChemiDoc (Bio-Rad, USA) gel documentation system. All Western blot experiments were performed using three independent biological replicates (*n* = 3). Representative immunoblots are shown in the figures, and densitometric quantification was performed using data from the three biological replicates.

### Pyroptosis assay

After 24 h of shNC or shRCN1 lentiviral transfection, NB4 cells were subjected to puromycin selection (2 µg·mL^-1^) under conditions with or without 0.5 µΜ STING inhibition (HY-112693; MedChemExpress). Flow cytometry was performed to detect cleaved caspase-1 using a FLICA® 660 Caspase-1 Assay Kit (9122; Immunochemistry)^[11]^.

### Immunoprecipitation-mass spectrometry

Immunoprecipitation (IP) of RCN1 was performed in NB4 cells using a Pierce mass spectrometry (MS)-compatible magnetic IP kit (90409; Thermo Fisher Scientific). Fifty milligrams of the cellular pellet was lysed with 500 μL of immunoprecipitation combined with mass spectrometry (IP-MS) cell lysis buffer following the supplier’s protocol. The lysates were then mixed with 5 μg of IP antibody: anti-RCN1 (ab1989996; Abcam) or a rabbit IgG1 control (ab172730; Abcam) per sample at 4 °C overnight, followed by capture of the immune complexes using MS magnetic beads for 1 h at room temperature with continuous mixing on a rotating platform. Next, the immunoprecipitated beads were washed with IP-MS wash buffer, then eluted with IP-MS elution buffer. The eluted samples were subjected to MS analysis at Jingjie PTM BioLab (Suzhou, China).

### Co-IP

A Capturem Co-IP kit (635721, Takara Bio, USA) was used to prepare the samples and perform the IP according to the manufacturer’s protocol. Cell lysate (500 μL) was incubated with 5 μg of antibodies against RCN1, ATAD3A, or TFAM, or with rabbit IgG1 control (ab172730; Abcam). The mixture was applied to a pre-equilibrated Protein A column and centrifuged at 1,000 × *g* for 1 min at room temperature. After washing the column with 100 µL of wash buffer, bound complexes were eluted with 30 µL of elution buffer. The eluate was centrifuged at 1,000 × *g* for 1 min to collect the antibody-protein complexes, followed by a western blot analysis as described above. A sample corresponding to 2% of the input lysate was used as a control.

### Proximity ligation assays

A proximity ligation assay (PLA) was performed using a Duolink *In Situ* Red Starter Kit (DUO92008; Sigma-Aldrich) in accordance with the manufacturer’s protocol. Cells overexpressing Myc-tagged ATAD3A (HeLa-ATAD3A) were first fixed using 4% paraformaldehyde (PFA) (Thermo Fisher Scientific), followed by permeabilization with 0.1% Triton X-100 and overnight incubation at 4 °C with primary antibodies against RCN1 (1:200, ab1989996; Abcam), ATAD3A (1:100, H00055210-D01; Abnova), Myc tag (1:50, 60003-2-Ig; Proteintech), and TFAM (1:100, sc-376672; Santa Cruz Biotechnology) prepared in blocking buffer. Duolink PLA probes (Anti-Mouse MINUS, DUO92004; Anti-Rabbit PLUS, DUO92002; Sigma-Aldrich) were then added at a 1:5 dilution and incubated at 37 °C for 1 h. After washing, a ligation was performed for 30 min, followed by signal amplification for 100 min using a red detection reagent. Samples were mounted using Duolink *In Situ* Mounting Medium containing 4’,6-diamidino-2-phenylindole (DAPI). Confocal images were acquired on an LSM800 microscope (Zeiss, Germany) and quantified manually using the “Threshold” and “Analyze Particles” tools in ImageJ/Fiji.

### Confocal microscopy imaging

For cellular staining, HeLa and HeLa-ATAD3A cells were first seeded onto 12-mm poly-d-lysine-coated coverslips (Cytoglass, China) in 24-well plates at 5 × 10^4^ cells per well and cultured for 24 h. Cells were first fixed in 4% PFA at room temperature for 15 min, then treated with 0.1% Triton X-100 for permeabilization and incubated in phosphate-buffered saline (PBS) containing 5% FBS for blocking. Samples were incubated with primary antibodies, including anti-DNA (1:200, AC-30-10; Progene), anti-single-stranded DNA binding protein 1 (SSBP1, 1:1,000, 12212-1-AP; Proteintech), anti-RCN1 (1:1,000, ab1989996; Abcam), anti-ATAD3A (1:1,000, H00055210-D01; Abnova), anti-Myc tag (1:500, A190-104A; Thermo Fisher Scientific), and TFAM (1:1,000, sc-376672, Santa Cruz Biotechnology) overnight at 4 °C. After being washed, the cells were treated with Alexa Fluor 647-conjugated donkey anti-rabbit IgG (H+L) (711-605-152; Jackson ImmunoResearch) and Alexa Fluor 568-conjugated mouse anti-rabbit IgG (H+L) (715-575-150; Jackson ImmunoResearch) secondary antibodies for 1 h at room temperature. Alexa Fluor secondary antibodies were used at 1:500, and the coverslips were mounted with DAPI Fluoromount-G (36308ES; YEASEN, China). Confocal imaging was carried out on an LSM 800 microscope (Zeiss, Germany).

### RBCEV purification

RBCEVs were purified following a previously described method^[31]^. Peripheral blood (10 mL per donor) was collected from 10 healthy adult volunteers (6 males and 4 females, aged 24-55 years) with no history of chronic diseases or active infections. Collection of peripheral blood samples was approved by the Institutional Review Board of Shenzhen Second People’s Hospital (Approval No. 2023-194-02PJ). Written informed consent was obtained from all participants in accordance with the Declaration of Helsinki. Briefly, red blood cells (RBCs) were prepared from whole blood using a leukodepletion filter (Nigale, China). Vesiculation was induced by incubating RBCs in PBS supplemented with 0.1 mg·mL^-1^ CaCl_2_ and 10 μM calcimycin (HY-N6687; MedChemExpress), followed by overnight incubation at 37 °C in 5% CO_2_. Following vesiculation, cell debris and residual RBCs were removed by sequential centrifugation. The supernatant was subsequently subjected to ultracentrifugation to pellet RBCEVs. The EV pellet was resuspended in PBS and further purified by size-exclusion chromatography (SEC). Purified RBCEVs were collected, suspended in PBS supplemented with 4% D-(+)-Trehalose (HY-N1132; MedChemExpress), and maintained at -80 °C until use.

### RBCEV characterization

RBCEVs were processed for transmission electron microscopy (TEM) analysis by fixation in 2% PFA for 10 min at room temperature. Next, 20 μL of the fixed sample was applied dropwise onto 200-mesh grids and allowed to adsorb for 10 min at room temperature. Excess liquid was gently blotted away with filter paper, and the grids were subsequently treated with 2% uranyl acetate for 5 min to achieve negative staining, after which the remaining stain was removed. After air-drying for 10 min, samples were imaged using a JEM1400 transmission electron microscope (JEOL, Tokyo, Japan). RBCEV size distribution, particle concentration, and zeta potential were analyzed using a Zetaview PMX120-Z instrument (Particle Metrix, Meerbusch, Germany). Purified RBCEVs were diluted in 10 mM HEPES buffer to achieve an optimal particle concentration range (1 × 10⁷-1 × 10^8^ particles·mL^-1^) and loaded into a pre-calibrated sample chamber. Measurements were performed at 11 distinct positions per sample, and each sample was analyzed in triplicate. The instrument was calibrated using 100-nm polystyrene standard particles prior to analysis. All measurements were conducted at a controlled temperature of 23-25 °C.

### siRNA loading and quantitation

The siRNA, including siNC, siRCN1, and Cy5-labeled siRCN1, was commercially synthesized by BGI (Shenzhen, China) and subsequently loaded into RBCEVs via transfection. Following the manufacturer’s protocol, a total of 1 μg of siRNA was transfected into 50 μg of RBCEVs using Exo-Fect^TM^ Exosome Transfection Kit (System Biosciences, USA). Supplementary Table 3 summarizes the siRNA sequences used in this study. To remove the unencapsulated siRNA and transfection reagents, siRNA-loaded RBCEVs underwent three washes with PBS, followed by centrifugation (21,000 × *g*) for 30 min after each wash. To determine the efficiency of siRNA loading, the transfected RBCEV pellets were dissolved in 10 μL of nuclease-free water supplemented with 1% Triton X-100. Electrophoretic separation was performed on a 2% agarose gel after the lysate was treated with 6× DNA loading dye (New England Biolabs). A gradient dilution of free siRNA was used as a control. Gels were imaged using a Bio-Rad ChemiDoc gel documentation system.

### Cellular uptake assay

For a cellular uptake analysis of the siRNA-loaded RBCEVs, a total of 1 × 10^5^ THP-1 cells were seeded into each well and maintained overnight. The cells were treated for 2 h with 20 μg unlabeled or Cy5-labeled siRCN1-loaded RBCEVs. Cells were collected following treatment and subjected to two washes with washing buffer (PBS containing 1% FBS), and the proportion of Cy5-positive cells was analyzed by flow cytometry (Novios; Beckman Coulter, USA).

### Generation of AML xenograft model and *in vivo * treatment with siRNA-loaded RBCEVs

Female NOD scid gamma (NSG) mice (aged approximately 7 weeks) were obtained from the Shanghai Model Organisms Center (Shanghai, China). All animal procedures followed the National Institute of Health guidelines and were approved by the Institutional Animal Care and Use Committee of Shenzhen Following Precision Medical Research Institute (license no. AP-SZZX-2020-12-013). To establish an AML xenograft model, we subcutaneously injected 1 × 10^6^ THP-1 cells into the right flank of NSG mice. Tumor size was assessed every 3 days using a digital caliper. The corresponding volumes were determined through the following equation: V = ^1^/_2_ × length × width^2^ (mm^3^). Upon reaching a tumor volume of approximately 50 mm^3^, the tumor-bearing mice were randomly assigned to different treatment groups by simple randomization (*n* = 6 per group) and intratumorally injected with either siRCN1-loaded RBCEVs (siRCN1-EV) or scrambled negative control siRNA-loaded RBCEVs (siNC-EV) every 3 days for a total of four injections. Each injection contained 50 μg RBCEVs, resulting in a cumulative dose of 200 μg EVs per mouse. The siNC-EV group served as the negative control to account for any effects associated with the RBCEV carrier and nonspecific siRNA delivery. Humane endpoints included a tumor volume exceeding 1,500 mm^3^, ulceration or necrosis of the tumor, body weight loss greater than 20%, or signs of severe distress, impaired mobility, or inability to access food or water. Animals reaching these criteria were euthanized immediately. On day 12 after the first intratumoral injection, all mice were euthanized, and tumors, blood, and organs (including kidney, heart, spleen, and liver) were collected for subsequent analysis.

### Toxicity and biochemical assessment

Throughout the animal experiments, the mice were routinely monitored for signs of toxicity or stress. Upon completion of the treatment, blood samples were collected from mice under isoflurane anesthesia via retro-orbital bleeding. To evaluate potential treatment-related toxicity, biochemical analysis was performed using a Mindray BS-420 automatic biochemistry analyzer (Shenzhen, China), according to the manufacturer’s instructions. The following parameters were measured: alkaline phosphatase (ALP), aspartate aminotransferase (AST), alanine aminotransferase (ALT), creatinine, total bilirubin, and creatine kinase (CK).

### Hematoxylin and eosin (H&E) staining

At the end of the treatment, AML mouse organs, including the heart, liver, spleen, and kidney, were harvested and fixed overnight in 10% buffered formalin (Thermo Fisher Scientific). The tissues were dehydrated through a graded ethanol series (70%, 95%, and 100%) and embedded in paraffin (Thermo Fisher Scientific) following three baths in molten paraffin at 62 °C. Sections (5 μm thick) were prepared using a microtome (RM2135; Leica). The sections were dewaxed in xylene and rehydrated using a descending ethanol gradient (95%, 90%, 80%, and 70%). Subsequently, the sections were stained with a hematoxylin solution (H8070; Solarbio, China), rinsed with tap water, differentiated in 1% hydrochloric acid alcohol for several seconds, rinsed once more, and blued in 0.6% ammonia water. The sections were counterstained with eosin (E8080; Solarbio, China) for 3-5 min. After dehydration using an ascending ethanol series (70%, 95%, and 100%) and clearing in xylene, the sections were mounted with neutral balsam (G8590; Solarbio, China). Finally, the slides were scanned using a Panoramic 1000 scanner (3DHISTECH Ltd., Budapest, Hungary).

### Statistical analysis

GraphPad Prism 9 was used for statistical analysis. An unpaired two-tailed Student’s *t*-test was employed to compare data between the control and treated groups. For datasets involving more than two groups, one-way ANOVA followed by Tukey’s multiple comparisons test was used. Two-way analysis of variance (ANOVA) was used for intergroup comparisons. All experiments were performed in at least three independent replicates (*n* = 6 mice per group for the animal experiments). Statistical significance was set at *P* < 0.05. Data are shown as mean (standard deviation).

## RESULTS

### RCN1 downregulation activates cGAS-STING-type I interferon pathway

Our previous work suggested that RCN1 downregulation promotes pyroptosis via the type I interferon pathway, and that STING inhibition partially attenuates this effect, implicating engagement of the cGAS-STING pathway^[11]^; however, the detailed mechanism has not yet been fully elucidated. Compared with control cells, NB4 cells transfected with shRCN1 lentivirus exhibited increased pyroptosis, while treatment with a 0.05 μM STING-I attenuated the RCN1 downregulation-induced pyroptosis [Figure 1A]. STING-I also partially reversed the RCN1 deficiency-reduced NB4 cellularity [Figure 1B]. RCN1 deficiency markedly reduced the NB4 cellularity [Figure 1B], and similar effects were observed in THP-1 and OCI-AML3 cells [Figure 1C and D]. To determine whether RCN1 knockdown influences innate immune signaling, we assessed the cGAS-STING-type I interferon pathway in AML cell lines. Western blotting revealed increased expression of phosphorylated STING, phosphorylated TBK1, and OAS3 in RCN1-deficient NB4, THP-1, and OCI-AML3 cells [Figure 1E-G], indicating pathway activation. Densitometric analyses of the western blot results further confirmed these findings [Supplementary Figure 1A-C]. Furthermore, qPCR results showed significantly elevated levels of interferon-stimulated genes (ISGs), including *IFIT1*, *IFI27*, *RSAD2*, *OAS1*, and *ISG15*, in RCN1-knockdown cells compared to control cells [Figure 1H-J]. In summary, these results indicate that RCN1 downregulation activates the cGAS-STING-type I interferon pathway in AML cells.

**Figure 1:**
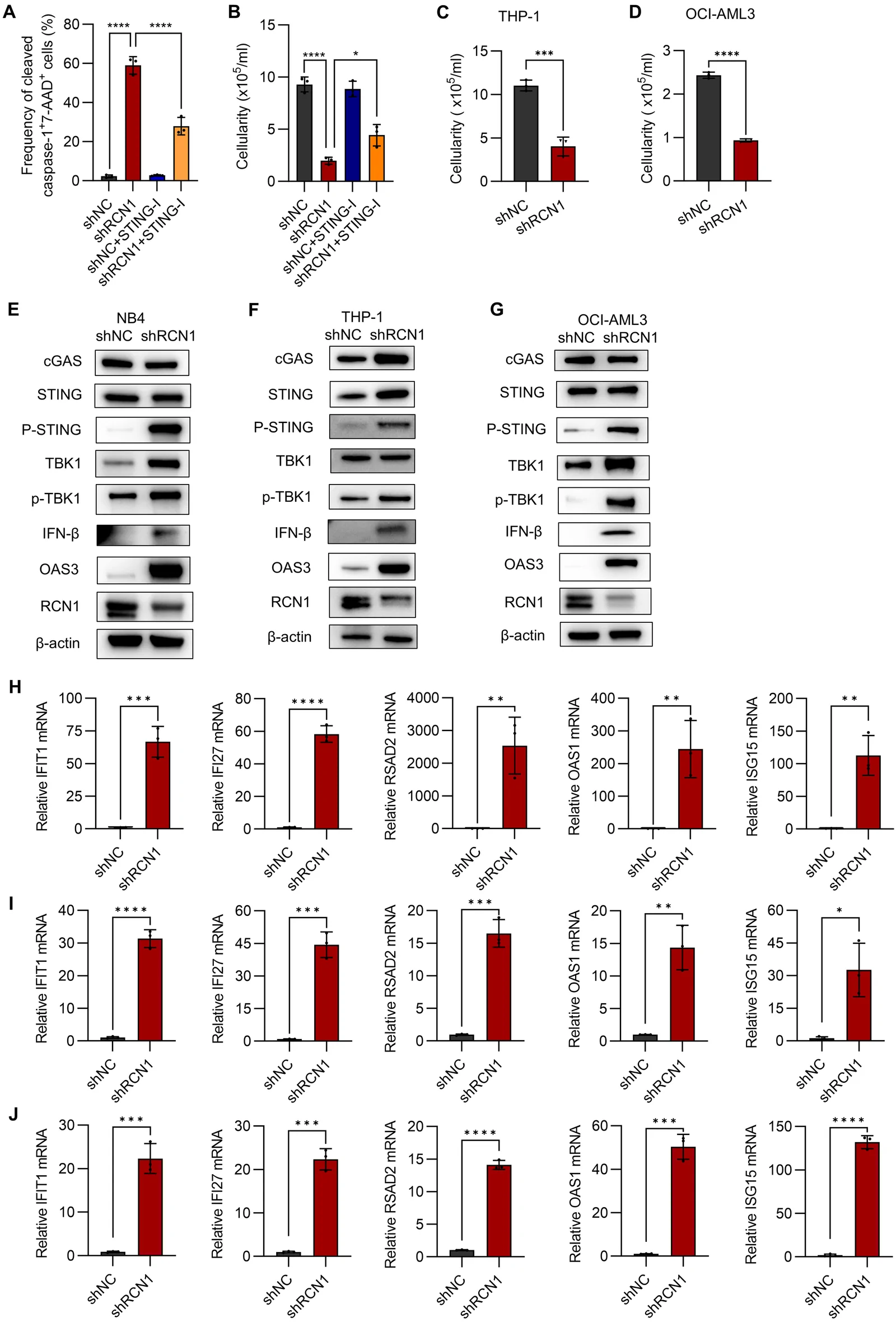
RCN1 knockdown is associated with cGAS-STING signaling in AML cells. (A) Pyroptotic cell death was assessed in NB4 cells transfected with shNC (*n* = 3) or shRCN1 lentiviruses (*n* = 3), with or without STING-I treatment. Measurements were performed on day 5 via the detection of active caspase-1 using a FLICA® 660 assay; (B) Cellularity of NB4 cells transfected with shNC (*n* = 3) or shRCN1 (*n* = 3) lentivirus and treated with or without STING-I on day 5; (C and D) Cellularity of THP-1 (C) and OCI-AML3 (D) cells following RCN1 deficiency; (E-G) Western blot detection of key proteins in the cGAS-STING-type I interferon pathway (including IFN-β and OAS3) in NB4 (E), THP-1 (F), and OCI-AML3 (G) cells treated with shNC or shRCN1 lentiviruses; (H-J) The mRNA expression levels of ISGs including *IFIT1*, *IFI27*, *RSAD2*, *OAS1*, and *ISG15* in RCN1-deficient NB4 (H), THP-1 (I), and OCI-AML3 (J) cells, as determined by qPCR. Results are shown as mean ± standard deviation. Statistical analyses were performed using one-way ANOVA followed by Tukey’s multiple comparisons test for panels involving more than two groups (A and B) and a two-tailed unpaired *t*-test for panels involving two-group comparisons (C and D, H-J). ^*^P < 0.05; ^**^P < 0.01; ^***^P < 0.001; ^****^P < 0.0001. AML: Acute myeloid leukemia; ANOVA: analysis of variance; cGAS: cyclic GMP-AMP synthase; FLICA: fluorescent-labeled inhibitor of caspases; *IFI27*: interferon alpha-inducible protein 27; *IFIT1*: interferon-induced protein with tetratricopeptide repeats 1; IFN-β: interferon beta; *ISG15*: interferon-stimulated gene 15; ISGs: interferon-stimulated genes; mRNA: messenger RNA; *OAS1*: 2’-5’-oligoadenylate synthetase 1; OAS3: 2’-5’-oligoadenylate synthetase 3; qPCR: quantitative polymerase chain reaction; RCN1: reticulocalbin 1; *RSAD2*: radical S-adenosyl methionine domain containing 2; shNC: short hairpin negative control; shRCN1: short hairpin RNA targeting RCN1; STING: stimulator of interferon genes; STING-I: STING inhibitor.

### RCN1 knockdown induces cGAS-STING signaling via mtDNA release

Disruption of mitochondrial integrity can lead to mtDNA escape into the cytosol. Given that cytoplasmic mtDNA activates cGAS-STING signaling, we initially measured cytosolic mtDNA levels using qPCR. The cytosolic mtDNA/nDNA ratio increased by approximately 1.63-fold in RCN1-deficient NB4 cells and approximately 1.7-fold in RCN1-deficient THP-1 cells [Figure 2A and B]. To determine whether mtDNA release contributes causally to cGAS-STING activation following RCN1 depletion, we next depleted mtDNA using ethidium bromide (EB) treatment and examined the resulting effects on downstream signaling. Low concentrations of EB reportedly inhibit mtDNA replication and transcription, leading to mitochondrial dysfunction and eventual mtDNA depletion while having little effect on nDNA. Compared to the controls, EB treatment markedly reduced mtDNA content by approximately 77% in RCN1-knockdown NB4 cells and approximately 82% in THP-1 cells [Figure 2C and D].

**Figure 2:**
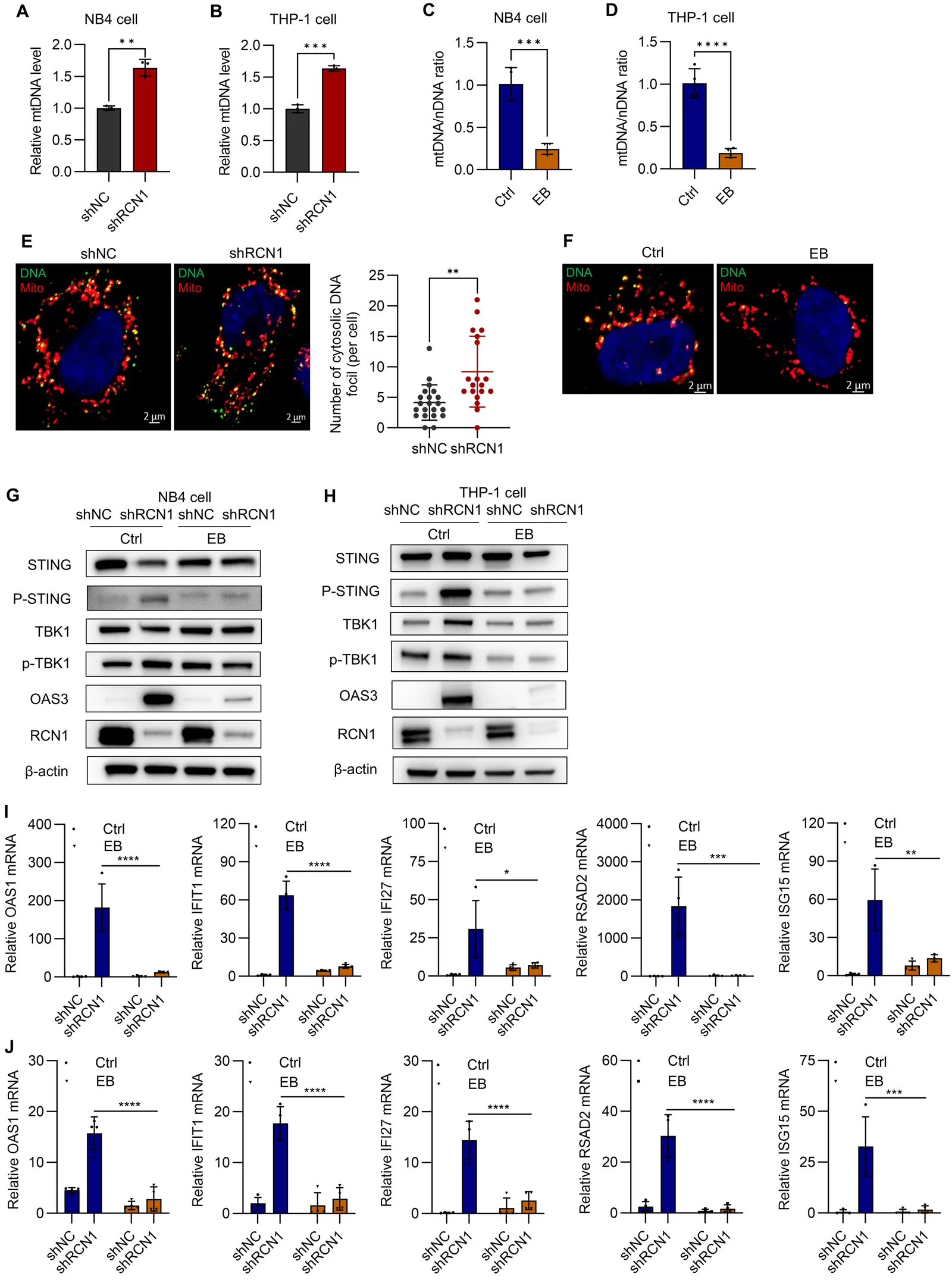
RCN1 deficiency promotes cGAS-STING-associated type I interferon signaling through mtDNA leakage. (A and B) Relative mtDNA levels in NB4 (A) and THP-1 (B) cells transfected with shNC (*n* = 3) or shRCN1 (*n* = 3) lentivirus as measured by qPCR; (C and D) The mtDNA content in EB-treated RCN1-deficient NB4 (C) and THP-1 (D) cells was determined by qPCR; (E) Representative confocal images of immunofluorescence staining of dsDNA (DNA, green), mitochondria (SSBP1, red), and nucleus (DAPI, blue) in HeLa cells transfected with shNC or shRCN1 lentiviruses. Co-localization of DNA and mitochondrial signals (mtDNA) is shown in yellow. The scale bars represent 2 μm. Quantification of DNA spots per cell that are not colocalized with mitochondrial markers is shown in the right graph (*n* = 21 for shNC, *n* = 19 for shRCN1); (F) Representative confocal images of immunofluorescence staining of dsDNA (DNA, green), mitochondria (SSBP1, red), and nucleus (DAPI, blue) in HeLa cells with or without EB treatment. DNA-mitochondria co-localization (mtDNA) is indicated in yellow. The scale bars represent 2 μm; (G and H) Western blot detecting the expression of proteins associated with the cGAS-STING-type I interferon signaling in RCN1-deficient NB4 (G) and THP-1 (H) cells under conditions with or without EB; (I and J) The mRNA level of ISGs (*OAS1*, *IFIT1*, *IFI27*, *RSAD2*, and *ISG15*) in RCN1-knockdown NB4 (I) and THP-1 (J) cells with or without EB treatment, as measured by qPCR. Data are expressed as mean ± standard deviation. Statistical significance was analyzed using two-way ANOVA, followed by Tukey’s multiple comparisons test for (I and J). For (A-D), statistical significance was assessed using a two-tailed unpaired *t*-test. ^*^P < 0.05; ^**^P < 0.01; ^***^P < 0.001; ^****^P < 0.0001. ANOVA: Analysis of variance; cGAS: cyclic GMP-AMP synthase; DAPI: 4’,6-diamidino-2-phenylindole; dsDNA: double-stranded DNA; EB: ethidium bromide; HeLa: human cervical cancer cell line; *IFI27*: interferon alpha-inducible protein 27; *IFIT1*: interferon-induced protein with tetratricopeptide repeats 1; *ISG15*: interferon-stimulated gene 15; ISGs: interferon-stimulated genes; mRNA: messenger RNA; mtDNA: mitochondrial DNA; *OAS1*: 2’-5’-oligoadenylate synthetase 1; qPCR: quantitative polymerase chain reaction; RCN1: reticulocalbin 1; *RSAD2*: radical S-adenosyl methionine domain containing 2; shNC: short hairpin negative control; shRCN1: short hairpin RNA targeting RCN1; SSBP1: single-stranded DNA-binding protein 1; STING: stimulator of interferon genes.

To directly visualize mtDNA redistribution following RCN1 depletion, we performed confocal microscopy in HeLa cells. RCN1 downregulation in HeLa cells led to an increased accumulation of cytosolic DNA foci that did not co-localize with mitochondrial signals as detected by confocal microscopy following dsDNA and mitochondrial staining, consistent with mtDNA release [Figure 2E]. Confocal microscopy consistently revealed a reduction in mtDNA foci, as indicated by decreased co-localization between DNA and mitochondrial signals, in EB-treated HeLa cells following dsDNA and mitochondrial staining [Figure 2F]. Moreover, EB treatment partially attenuated the cGAS-STING-type I interferon response in RCN1-knockdown NB4 and THP-1 cells [Figure 2G and H] and reduced ISG expression [Figure 2I and J]. Overall, these results suggest that cytosolic mtDNA contributes to cGAS-STING-dependent type I interferon signaling induced by RCN1 downregulation.

### RCN1 binds mitochondrial protein TFAM via mitochondrial scaffold protein ATAD3A

Since our findings suggested that RCN1 deficiency promotes mtDNA release and subsequent cGAS-STING activation, we next sought to identify the molecular mechanism linking RCN1 to mtDNA homeostasis. To this end, we performed IP-MS to identify proteins that may interact with RCN1 in wild-type NB4 cells. A bioinformatics analysis revealed that three mitochondrial proteins (ATAD3A, SSBP1, and TFAM) were among the top 10 differentially enriched proteins [Figure 3A and B]. We found that RCN1 deficiency was associated with reduced TFAM expression, with no apparent effect on ATAD3A or SSBP1 levels [Figure 3C]. Because TFAM plays a central role in maintaining mitochondrial nucleoid integrity and preventing mtDNA release, we focused our subsequent analyses on the potential relationship between RCN1 and TFAM. Given previous reports that ATAD3A serves as a key mitochondrial scaffold protein, TFAM is essential for maintaining mtDNA stability, and the ATPase domain of ATAD3A binds to TFAM^[33]^, we hypothesized that RCN1 downregulation induces mtDNA release by perturbing TFAM function via the ATAD3A scaffold. Co-IP assays using NB4 cells overexpressing RCN1 confirmed that ATAD3A bridges the interaction between RCN1 and TFAM [Figure 3D]. To further assess the intracellular association among these proteins, we performed a PLA to examine the interactions between endoplasmic reticulum (ER)-associated RCN1, mitochondrial ATAD3A, and TFAM. The PLA results further supported this finding. Red punctate signals indicated prominent intracellular interactions between ATAD3A and TFAM as well as between RCN1 and ATAD3A, whereas interactions between RCN1 and TFAM were reduced [Figure 3E]. Samples processed without primary antibodies showed no detectable signals and were used as negative controls (NC). Confocal microscopy further validated these findings. Co-localization signals (shown in yellow) were strong between ATAD3A and TFAM, detectable between RCN1 and ATAD3A, and reduced between RCN1 and TFAM [Figure 3F].

**Figure 3:**
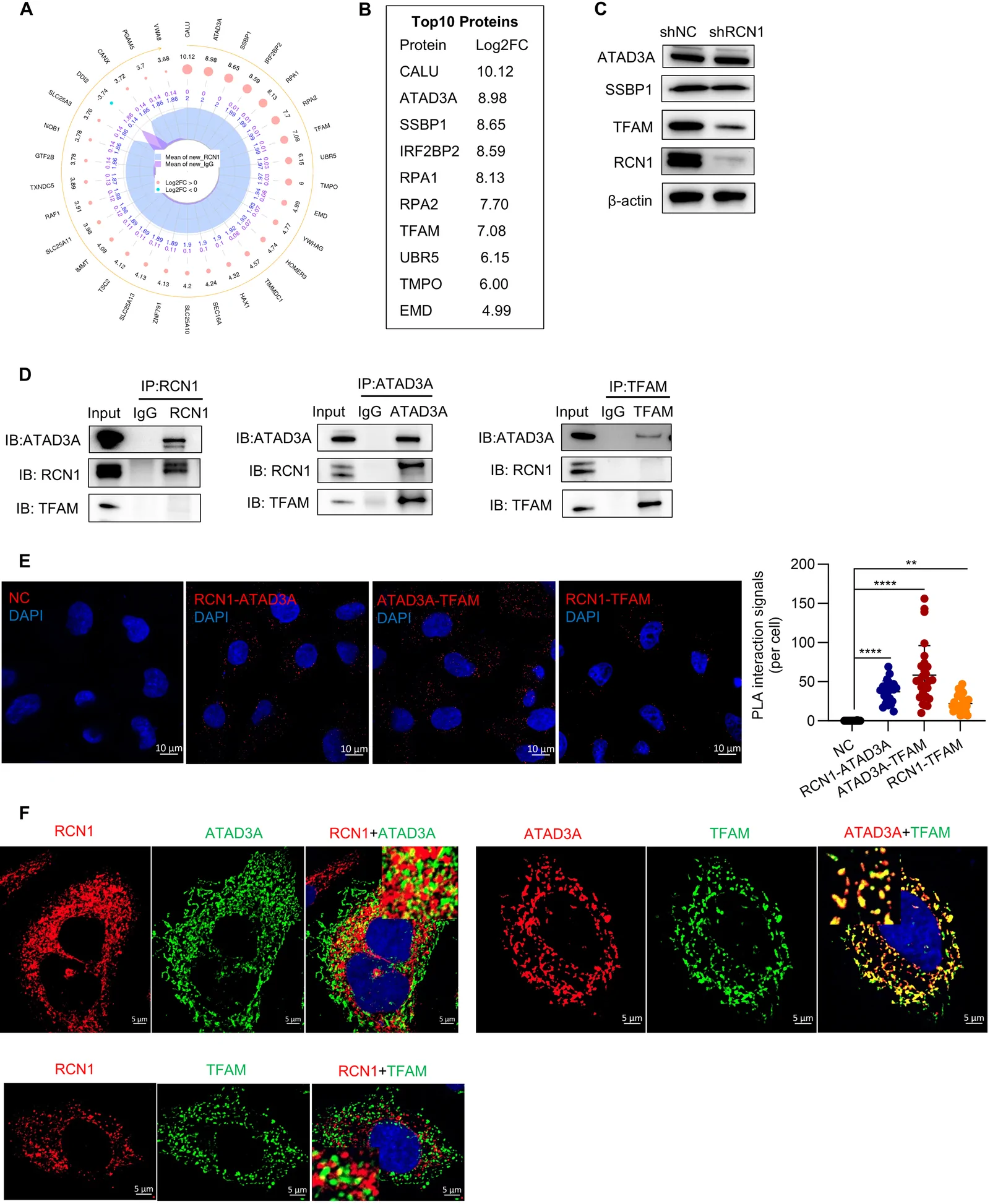
RCN1 interacts with the mitochondrial protein TFAM via the scaffold protein ATAD3A. (A) Radar chart displaying the top 30 differentially expressed proteins identified by IP-MS, ranked clockwise by the magnitude of the log_2_ fold change; (B) The top 10 differentially expressed proteins were sorted by the magnitude of the log_2_ fold change; (C) Protein expression levels of ATAD3A, SSBP1, and TFAM in RCN1-knockdown NB4 cells were analyzed by Western blot; (D) Co-IP assays in NB4 cells overexpressing RCN1 demonstrate that RCN1 associates with TFAM indirectly through ATAD3A; (E) Representative confocal images of an *in situ* PLA performed in HeLa-ATAD3A cells. The scale bar indicates 10 μm. A quantitative analysis of PLA signals is shown in the right panel corresponding to RCN1-ATAD3A, ATAD3A-TFAM, and RCN1-TFAM interactions per cell (*n* = 24 for RCN1-ATAD3A, *n* = 27 for ATAD3A-TFAM, and *n* = 26 for RCN1-TFAM); (F) Representative confocal microscopy of HeLa-ATAD3A cells stained for RCN1 (red), ATAD3A (red or green), TFAM (green), and nuclei (DAPI, blue). The scale bars indicate 5 μm. The insets show higher-magnification views of the indicated regions. Data are expressed as mean ± standard deviation. Statistical significance was assessed using one-way ANOVA followed by Tukey’s multiple comparisons test. ^**^P < 0.01; ^****^P < 0.0001. ANOVA: Analysis of variance; ATAD3A: ATPase family AAA domain-containing protein 3A; Co-IP: co-immunoprecipitation; DAPI: 4’,6-diamidino-2-phenylindole; HeLa: human cervical cancer cell line; IB: immunoblot; IgG: immunoglobulin G; IP: immunoprecipitation; IP-MS: immunoprecipitation-mass spectrometry; PLA: proximity ligation assay; RCN1: reticulocalbin 1; SSBP1: single-stranded DNA-binding protein 1; TFAM: mitochondrial transcription factor A.

### TFAM links RCN1 downregulation to cGAS-STING-type I interferon signaling

Given that RCN1 deficiency reduced TFAM expression and that TFAM is a key regulator of mtDNA stability, we next investigated whether TFAM mediates the activation of cGAS-STING signaling induced by RCN1 downregulation. Compared with control cells, NB4 and OCI-AML3 cells infected with shTFAM showed decreased cell viability to approximately 63% and 51%, respectively [Figure 4A and B]. The cytosolic mtDNA levels increased by approximately 2-fold in TFAM-deficient NB4 cells and approximately 1.7-fold in TFAM-deficient OCI-AML3 cells [Figure 4C and D]. A western blot analysis further revealed enhanced cGAS-STING-type I interferon signaling in TFAM-knockdown cells, as reflected by elevated levels of phosphorylated STING, TBK1, and OAS3 [Figure 4E and F]. Notably, when TFAM was concurrently overexpressed with RCN1 knockdown in NB4 cells, TFAM upregulation reversed the phenotypic changes induced by RCN1 downregulation, including mtDNA leakage, reduced cell viability, and type I interferon pathway activation [Figure 4G-J]. Taken together, these results demonstrate that RCN1 downregulation triggers cGAS-STING-type I interferon signaling through TFAM dysregulation.

**Figure 4:**
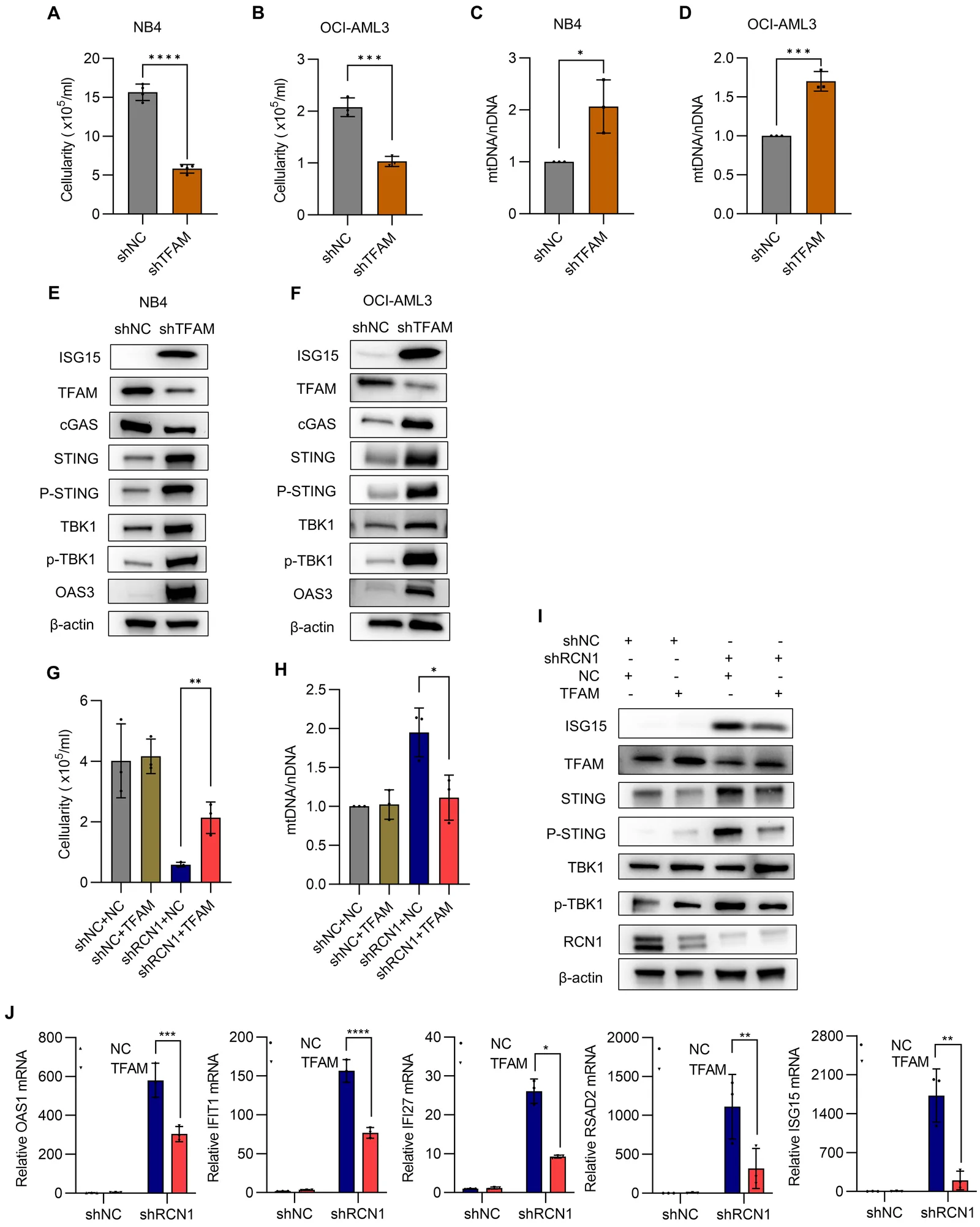
TFAM mediates cGAS-STING pathway activation induced by RCN1 deficiency. (A and B) Cellularity of NB4 (A) or OCI-AML3 (B) cells infected with shNC (*n* = 3) or shTFAM (*n* = 3) lentiviruses; (C and D) Relative cytosolic mtDNA levels in NB4 (C) or OCI-AML3 (D) cells infected with shNC (*n* = 3) or shTFAM (*n* = 3) lentiviruses measured by qPCR; (E and F) Expression of cGAS-STING pathway proteins, IFN-β, and OAS3 in TFAM-knockdown NB4 (E) or OCI-AML3 (F) cells; (G) Changes in cellularity after TFAM overexpression in RCN1-deficient NB4 cells; (H) Cytosolic mtDNA levels in RCN1-deficient NB4 cells following TFAM overexpression were analyzed by qPCR; (I) Expression of cGAS-STING-related proteins in RCN1-deficient NB4 cells following TFAM upregulation; (J) Impact of TFAM overexpression on ISGs (*OAS1*, *IFIT1*, *IFI27*, *RSAD2*, and *ISG15*) mRNA levels in RCN1-knockdown NB4 cells measured via qPCR. Data are presented as mean ± standard deviation. Statistical significance was assessed using a two-tailed unpaired *t*-test. ^*^P < 0.05; ^**^P < 0.01; ^***^P < 0.001; ^****^P < 0.0001. cGAS: Cyclic GMP-AMP synthase; *IFI27*: interferon alpha-inducible protein 27; *IFIT1*: interferon-induced protein with tetratricopeptide repeats 1; IFN-β: interferon beta; *ISG15*: interferon-stimulated gene 15; ISGs: interferon-stimulated genes; mRNA: messenger RNA; mtDNA: mitochondrial DNA; *OAS1*: 2’-5’-oligoadenylate synthetase 1; OAS3: 2’-5’-oligoadenylate synthetase 3; qPCR: quantitative polymerase chain reaction; RCN1: reticulocalbin 1; *RSAD2*: radical S-adenosyl methionine domain containing 2; shNC: short hairpin negative control; shTFAM: short hairpin RNA targeting TFAM; STING: stimulator of interferon genes; TFAM: mitochondrial transcription factor A.

### RBCEV purification and characterization

Having identified RCN1 as a potential therapeutic target in AML, we next explored whether RCN1 silencing could be achieved using an RBCEV-mediated siRNA delivery strategy. Given their favorable biocompatibility, high nucleic acid-loading capacity, and biodistribution profile that overlaps with major sites of AML involvement, RBCEVs were selected as delivery vehicles. RBCEVs were isolated from peripheral blood samples donated by healthy volunteers after informed consent using a series of centrifugation steps, followed by SEC [Figure 5A]. Western blot verified the expression of vesicle-specific markers, including tumor susceptibility gene 101, stomatin, glycophorin A, and ALG-2-interacting protein X, whereas the ER marker calnexin was absent. Hemoglobin A and GAPDH, which were highly expressed in both RBCEVs and their parent blood cells, were also detected [Figure 5B]. A ZetaView analysis showed that the average RBCEV diameter was approximately 160 nm [Figure 5C]. Zeta potential measurements using ZetaView indicated a negative surface charge averaging -21 mV [Figure 5D]. TEM images revealed intact RBCEVs with a typical vesicle-like morphology and bilayer membrane structure [Figure 5E]. Similar results were obtained in two additional independent RBCEV preparations [Supplementary Figure 2A-C], indicating low batch-to-batch variability and good reproducibility of RBCEV production. To enable functional studies of RCN1 silencing, RBCEVs were subsequently loaded with siRNA using the Exo-Fect reagent. Agarose gel electrophoresis confirmed a loading efficiency of approximately 80% and was calibrated against a standard curve of free siRNA [Figure 5F].

**Figure 5:**
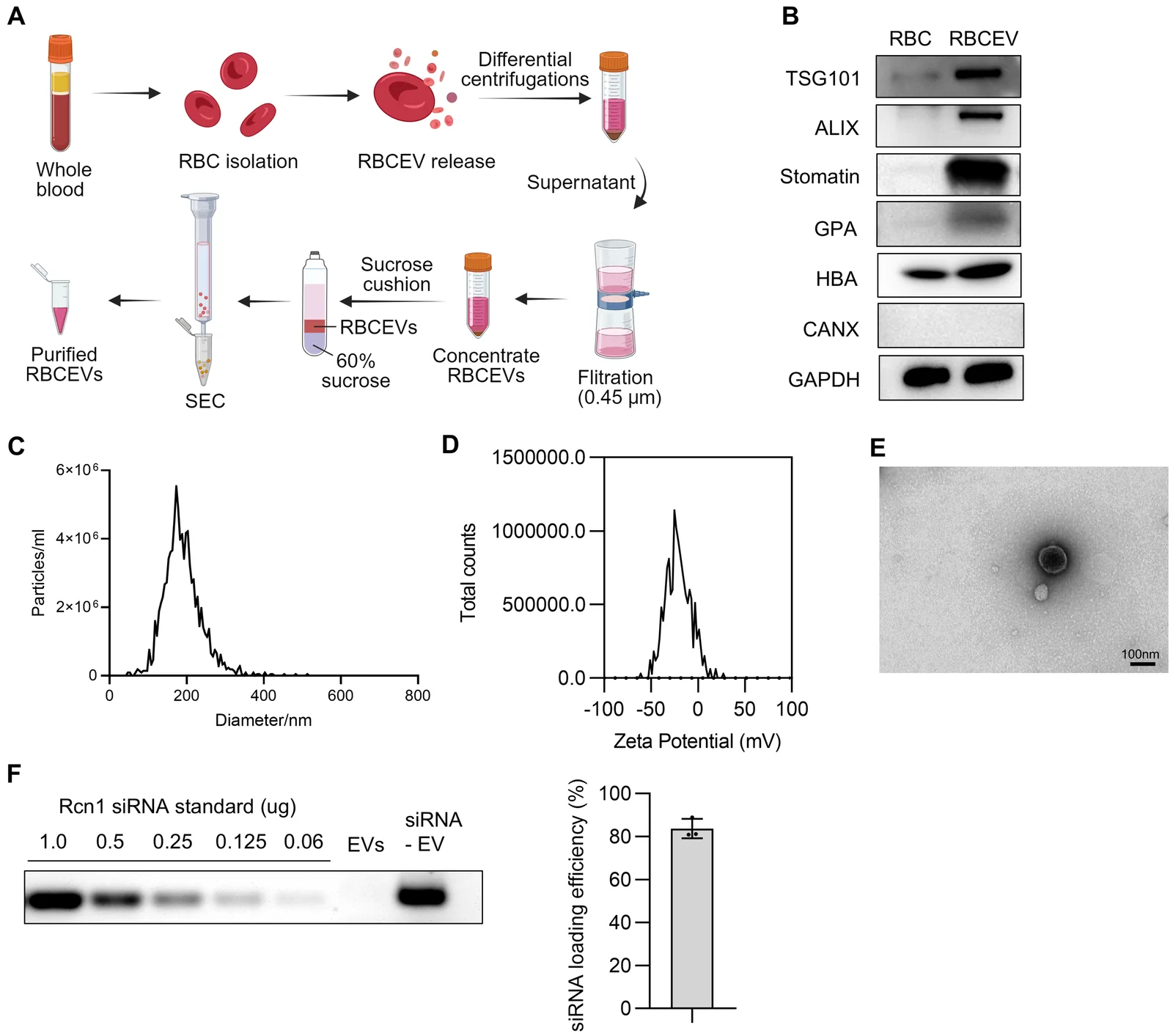
Characterization and siRNA loading efficiency of RBCEVs. (A) Schematic diagram of the RBCEV isolation procedure. Created in BioRender. Chen, H. (2026); (B) Western blot detection of marker proteins in purified RBCEVs (ALIX, GAPDH, GPA, HBA, and TSG101); (C) RBCEV particle size profile detected by ZetaView; (D) ZetaView analysis revealing the RBCEV zeta potential; (E) TEM revealed the morphology of RBCEVs. The scale bar indicates 100 nm. One of the three independent experiments is shown; (F) Quantification of the loading efficiency of siRNA-EV. Data are presented as the mean ± standard deviation. ALIX: ALG-2-interacting protein X; CANX: calnexin; EV: extracellular vesicle; GAPDH: glyceraldehyde-3-phosphate dehydrogenase; GPA: glycophorin A; HBA: hemoglobin A; RBC: red blood cell; RBCEV: red blood cell extracellular vesicle; RBCEVs: red blood cell extracellular vesicles; SEC: size-exclusion chromatography; siRNA: small interfering RNA; siRNA-EV: siRNA-loaded RBCEV; TEM: transmission electron microscopy; TSG101: tumor susceptibility gene 101.

### Targeting RCN1 significantly suppresses subcutaneous THP-1 xenograft growth

Upon confirming the loading of siRCN1 into RBCEVs, we first evaluated whether RBCEVs could efficiently deliver siRCN1 into AML cells. Cellular uptake of siRNA-loaded RBCEVs was assessed by incubating THP-1 cells with unlabeled (unlabeled-siRCN1-loaded EV) or Cy5-labeled siRCN1-loaded RBCEVs (Cy5-siRCN1-loaded EV), followed by flow cytometry. We detected up to approximately 95% Cy5-positive cells, indicating high cellular uptake efficiency [Figure 6A]. Notably, THP-1 cells used in this study were maintained in an undifferentiated state and therefore did not exhibit a macrophage-like phenotype. Undifferentiated THP-1 cells showed only modestly higher RBCEV uptake than NB4 and OCI-AML3 cells; PMA-induced macrophage-like THP-1 cells displayed markedly greater RBCEV uptake [Supplementary Figure 3]. Importantly, Cy5-siRNA loading did not noticeably affect the size distribution or morphology of RBCEVs, indicating that EV integrity was preserved [Supplementary Figure 4]. Moreover, free Cy5 dye and free Cy5-siRNA controls confirmed that the detected intracellular fluorescence was primarily attributable to RBCEV-mediated delivery of encapsulated siRNA rather than nonspecific uptake [Supplementary Figure 5]. Having confirmed efficient cellular uptake, we next examined whether RBCEV-mediated delivery resulted in effective RCN1 silencing and anti-leukemic activity. THP-1 cells were then treated with siRCN1-loaded RBCEVs (siRCN1-EV), which significantly reduced *RCN1* expression by approximately 67% and cell viability by approximately 54% compared with siNC-loaded RBCEVs (siNC-EV) [Figure 6B-D]. To explore the *in vivo* therapeutic effects of RBCEVs delivering siRCN1, an AML xenograft model was established by subcutaneously inoculating NSG mice with 1 × 10^6^ THP-1 cells. Mice received intratumoral injections every 3 days. Each injection contained 50 μg RBCEVs (total EV protein quantified by BCA assay), corresponding to an estimated 5.1 × 10^10^ particles based on the experimentally determined relationship between EV protein content and particle number [Supplementary Figure 6]. According to the experimentally determined siRNA loading efficiency (~80%), each RBCEV dose contained approximately 0.8 μg encapsulated siNC or siRCN1. The tumor size was measured with a caliper at 3-day intervals [Figure 6E]. Mice treated with siRCN1-EV exhibited significantly smaller tumors and slower leukemia growth than control mice [Figure 6F and G]. No statistically significant differences in body weight were detected during the treatment period [Figure 6H].

**Figure 6:**
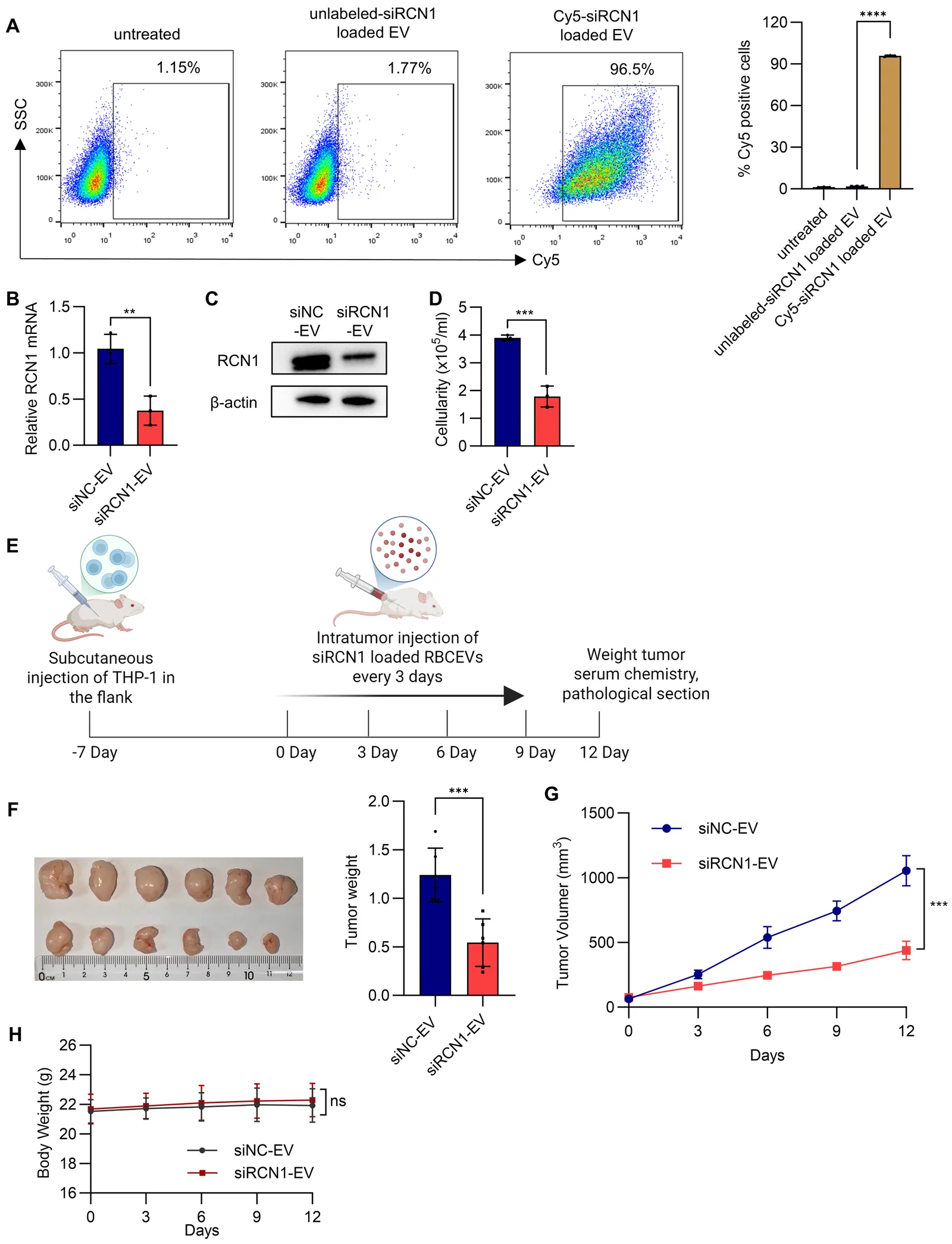
RBCEVs deliver siRCN1 to inhibit AML cell growth. (A) Flow cytometry analysis of Cy5-positive THP-1 cells following exposure to unlabeled or Cy5-labeled siRCN1-loaded RBCEVs; (B) RCN1 mRNA abundance in THP-1 cells following treatment with RBCEVs loaded with siNC or siRCN1, with normalization to *GAPDH*; (C) Western blotting was performed to detect RCN1 abundance in THP-1 cells after exposure to siNC- or siRCN1-loaded RBCEVs; (D) Viability of THP-1 cells after exposure to siNC- or siRCN1-loaded RBCEVs (*n* = 3); (E) Experimental timeline of RBCEV-based treatment in AML xenograft model. Created in BioRender. Chen, H. (2026); (F) Representative tumor images and tumor weights from xenograft mice receiving siNC- or siRCN1-loaded RBCEVs every 3 days (*n* = 6 per group); (G) Tumor volume was monitored at 3-day intervals during the treatment period; (H) Body weight was tracked throughout treatment to evaluate potential systemic toxicity. Data are expressed as mean ± standard deviation. Statistical significance was assessed using a two-tailed unpaired *t*-test (A and B, D and F) and a two-way ANOVA (G). ns, not significant. ^**^P < 0.01; ^***^P < 0.001; ^****^P < 0.0001. AML: Acute myeloid leukemia; ANOVA: analysis of variance; Cy5: cyanine 5; *GAPDH*: glyceraldehyde-3-phosphate dehydrogenase; mRNA: messenger RNA; RBCEV: red blood cell extracellular vesicle; RBCEVs: red blood cell extracellular vesicles; RCN1: reticulocalbin 1; siNC: small interfering negative control; siRCN1: small interfering RNA targeting RCN1.

### SiRCN1-loaded RBCEVs exhibit no detectable toxicity in AML treatment

At the end of treatment, whole blood, along with the liver, heart, spleen, and kidney, was obtained from mice to evaluate the potential toxicity of siRCN1-EV. Compared with the siNC-EV group, mice treated with siRCN1-EV showed no significant differences in kidney function markers (urea and creatinine) or the muscle damage marker CK [Figure 7A]. Because RBCEVs are prone to accumulation within the liver, potential hepatotoxicity was also assessed. No significant differences were observed in liver toxicity parameters, including ALP, AST, ALT, and total bilirubin levels, between the siRCN1-EV treatment group and the siNC-EV control group [Figure 7B]. Furthermore, H&E-stained sections of liver, heart, spleen, and kidney revealed no signiﬁcant pathologic changes in siNC-EV or siRCN1-EV treated mice compared with tumor-free mice, indicating that RBCEV-mediated delivery of siRCN1 is well-tolerated and non-toxic [Figure 7C-F].

**Figure 7:**
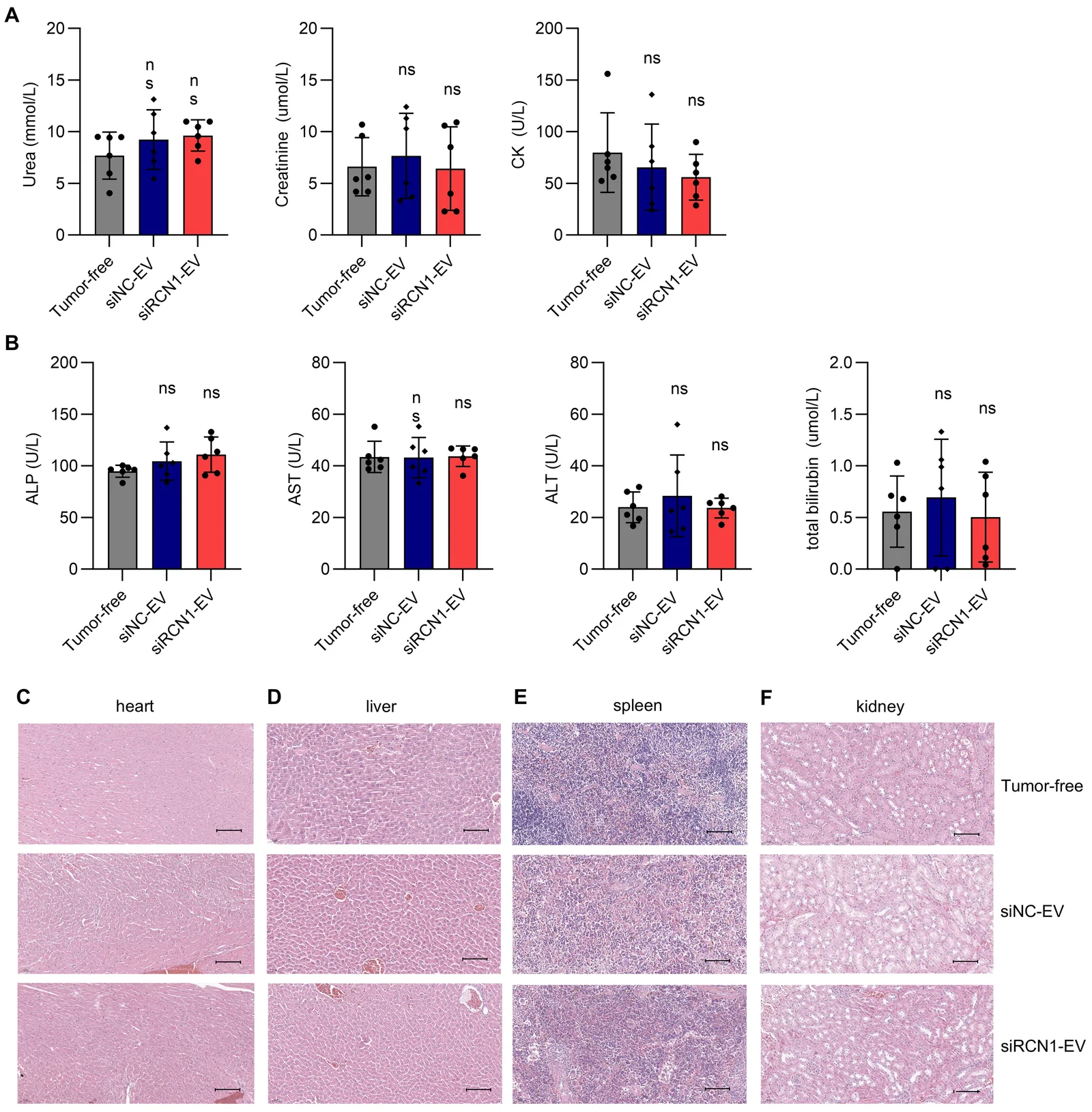
RBCEV-mediated delivery of siRCN1 demonstrates safety in AML treatment. (A) Kidney function markers (creatinine and urea) and muscle damage marker (CK) in mice treated with siNC- or siRCN1-loaded RBCEVs (*n* = 6); (B) Hepatotoxicity markers (total bilirubin, ALT, AST, and ALP) following treatment with siRNA-loaded RBCEVs; (C-F) Histological examination of H&E-stained heart (C), liver (D), spleen (E), and kidney (F) from tumor-free mice and siRNA-loaded RBCEV-treated mice. Scale bars represent 100 μm. Data are expressed as mean ± standard deviation. Statistical significance was assessed using a two-tailed unpaired *t*-test. ns, not significant. ALP: Alkaline phosphatase; ALT: alanine aminotransferase; AML: acute myeloid leukemia; AST: aspartate aminotransferase; CK: creatine kinase; H&E: hematoxylin and eosin; RBCEV: red blood cell extracellular vesicle; RBCEVs: red blood cell extracellular vesicles; RCN1: reticulocalbin 1; siNC: small interfering negative control; siRNA: small interfering RNA; siRCN1: small interfering RNA targeting RCN1.

## DISCUSSION

RCN1 is a Ca^2+^-binding protein that features six conserved EF-hand Ca^2+^-binding domains and an ER retention signal called HDEL (His-Asp-Glu-Leu). In addition to its involvement in calcium homeostasis and regulation of ER stress-induced apoptosis, elevated RCN1 expression has been reported across multiple cancer types and is closely linked to tumorigenesis, invasion, unfavorable prognosis, and drug resistance^[11]^. RCN1 downregulation suppresses cell proliferation and induces apoptosis, highlighting its potential as a target for tumor treatment^[8]^. Our previous work showed that patients with AML exhibit significant upregulation of *RCN1*, and RCN1 knockdown in AML primary cells and cell lines significantly suppressed proliferation while promoting type I interferon production and pyroptosis^[11]^. However, the mechanism by which RCN1 downregulation promotes type I interferon production remains unclear. Here, we showed that RCN1 knockdown was associated with increased cytoplasmic mtDNA levels. EB treatment reduced cytoplasmic mtDNA levels and attenuated cGAS-STING signaling triggered by RCN1 knockdown. This suggests that RCN1 downregulation promotes mtDNA escape into the cytosol, thereby initiating the cGAS-STING-type I interferon signaling axis. Mechanistically, TFAM, a key mitochondrial protein involved in mtDNA maintenance, is regulated by RCN1 through ATAD3A. RCN1 knockdown reduced TFAM expression, whereas restoration of TFAM expression rescued the phenotype caused by the RCN1 knockdown. Accordingly, we speculate that RCN1 binds and regulates TFAM and that its downregulation reduces TFAM expression, destabilizes mtDNA, and induces mtDNA leakage, thereby activating the cGAS-STING-type I interferon signaling pathway.

We identified a regulatory axis in which the ER-resident protein RCN1 influences the expression and function of TFAM, linking ER and mitochondrial homeostasis. Organelle contact sites, although occupying only a limited portion of the membrane surface, play essential roles in intracellular communication^[33]^. The mitochondria-associated ER membrane (MAM) is a well-characterized organelle contact site. ATAD3A is a mitochondrial scaffold protein of the AAA+ superfamily enriched at the MAM and implicated in ER-mitochondria communication^[34]^. In addition to interacting with ER stress-related proteins, ATAD3A associates with mitochondrial nucleoid components such as TFAM and contributes to mitochondrial dynamics and mtDNA organization^[35-37]^. Therefore, beyond the established ATAD3A/PERK and ATAD3A/GRP78/WASF3 signaling axes, we propose that the RCN1/ATAD3A/TFAM axis represents a novel pathway for ER-mitochondria communication.

Importantly, although lentiviral-mediated RCN1 silencing demonstrated antileukemic efficacy in our previous study^[11]^, viral vectors carry potential risks including genomic integration and sustained transgene expression, which may limit translational applicability. In contrast, EV-mediated delivery of siRNA provides a transient and non-integrative strategy that may offer improved safety and controllability. By employing RBCEVs as delivery vehicles, we achieved effective RCN1 silencing *in vivo* without detectable systemic toxicity, thereby enhancing the translational feasibility of targeting RCN1 in AML.

Given the complexity, heterogeneity, and dysregulation of multiple signaling pathways in cancer, combination siRNA therapy targeting distinct oncogenes may offer greater efficacy than single-target approaches^[38]^. Moreover, the abundance of undruggable targets limits conventional therapeutic options. The sequence-specific nature of siRNA therapeutics expands the range of therapeutically targetable genes and offers opportunities to modulate previously inaccessible genes^[39]^. Collectively, our findings suggest that RBCEVs represent a promising platform for RNA-based therapeutic delivery in AML and potentially other malignancies.

Nevertheless, several limitations of the present study should be acknowledged. First, the current study primarily relied on established AML cell lines and immunodeficient xenograft models. Although these models are widely used for mechanistic and preclinical investigations, they do not fully recapitulate the genetic heterogeneity, bone marrow niche, and immune microenvironment of human AML. Therefore, validation in primary AML samples, patient-derived xenograft models, and immunocompetent systems will be important to further establish the clinical relevance of the findings. Second, the therapeutic efficacy of RBCEV-delivered siRCN1 was evaluated using intratumoral administration. Although this approach enabled proof-of-concept validation of the therapeutic strategy, it does not fully reflect the clinical treatment setting of AML, a disseminated hematological malignancy. Future studies investigating systemic administration, biodistribution, pharmacokinetics, and long-term safety will be important for future clinical translation. In addition, although RBCEVs exhibited favorable biocompatibility and delivery efficiency in the present study, further optimization of the delivery platform may be required to maximize its therapeutic potential. The siRNA cargo used in the animal study was not chemically modified; therefore, its stability, circulation time, and resistance to nuclease-mediated degradation may be further improved through established modification strategies, such as 2’-O-methyl or phosphorothioate substitutions. Moreover, while RBCEVs demonstrated efficient uptake by AML cells, their delivery specificity could potentially be enhanced through the incorporation of targeting ligands, including peptides, antibodies, or nanobodies directed against AML-associated surface markers. Such modifications may reduce off-target uptake by normal tissues and improve accumulation at disease sites. Addressing these limitations will further strengthen the mechanistic understanding and translational potential of RCN1-targeted therapeutic strategies in AML.

## DECLARATIONS

### Availability of data and materials

The relevant data and materials for this study are available from the corresponding author (Zhang Q) upon reasonable request.

### Copyright

© The Author(s) 2026.
