# Supplementary material for: Extracellular vesicle-delivered siRNA targeting RCN1 suppresses acute myeloid leukemia through TFAM-dependent mtDNA-cGAS-STING signaling
Source: Extracell Vesicles Circ Nucl Acids. 2026 Jul 31;7(3):1228–48. doi: 10.20517/evcna.2026.36 (PMC13673257; doi:10.20517/evcna.2026.36)
Supplement: Supplementary file 1 [file evcna-7-3-1228-SupplementaryMaterials.pdf]

Supplementary Materials

Extracellular vesicle-delivered siRNA targeting RCN1 suppresses acute myeloid leukemia through TFAM-dependent mtDNA-cGAS-STING signaling

Huan Chen[1], Na An[1], Linlin Yang[1], Jin Lou[1], Yuming Pan[1], Minh T.N. Le[2], Xin Du[1], Qiaoxia Zhang[1]

[1]Shenzhen Bone Marrow Transplantation Public Service Platform, Shenzhen Institute of Hematology, Shenzhen Second People's Hospital, First Affiliated Hospital of Shenzhen University, Shenzhen University Health Sciences Center, Shenzhen 518025, Guangdong, China.
[2]Institute for Digital Medicine and Department of Pharmacology, Yong Loo Lin School of Medicine, National University of Singapore, Singapore 117600, Singapore.

Correspondence to: Prof. Qiaoxia Zhang, Prof. Xin Du, Shenzhen Bone Marrow Transplantation Public Service Platform, Shenzhen Institute of Hematology, Shenzhen Second People's Hospital, First Affiliated Hospital of Shenzhen University, Shenzhen University Health Sciences Center, Shenzhen 518025, Guangdong, China. E-mail: qiaoxiazhang@email.szu.edu.cn; duxin2023@email.szu.edu.cn

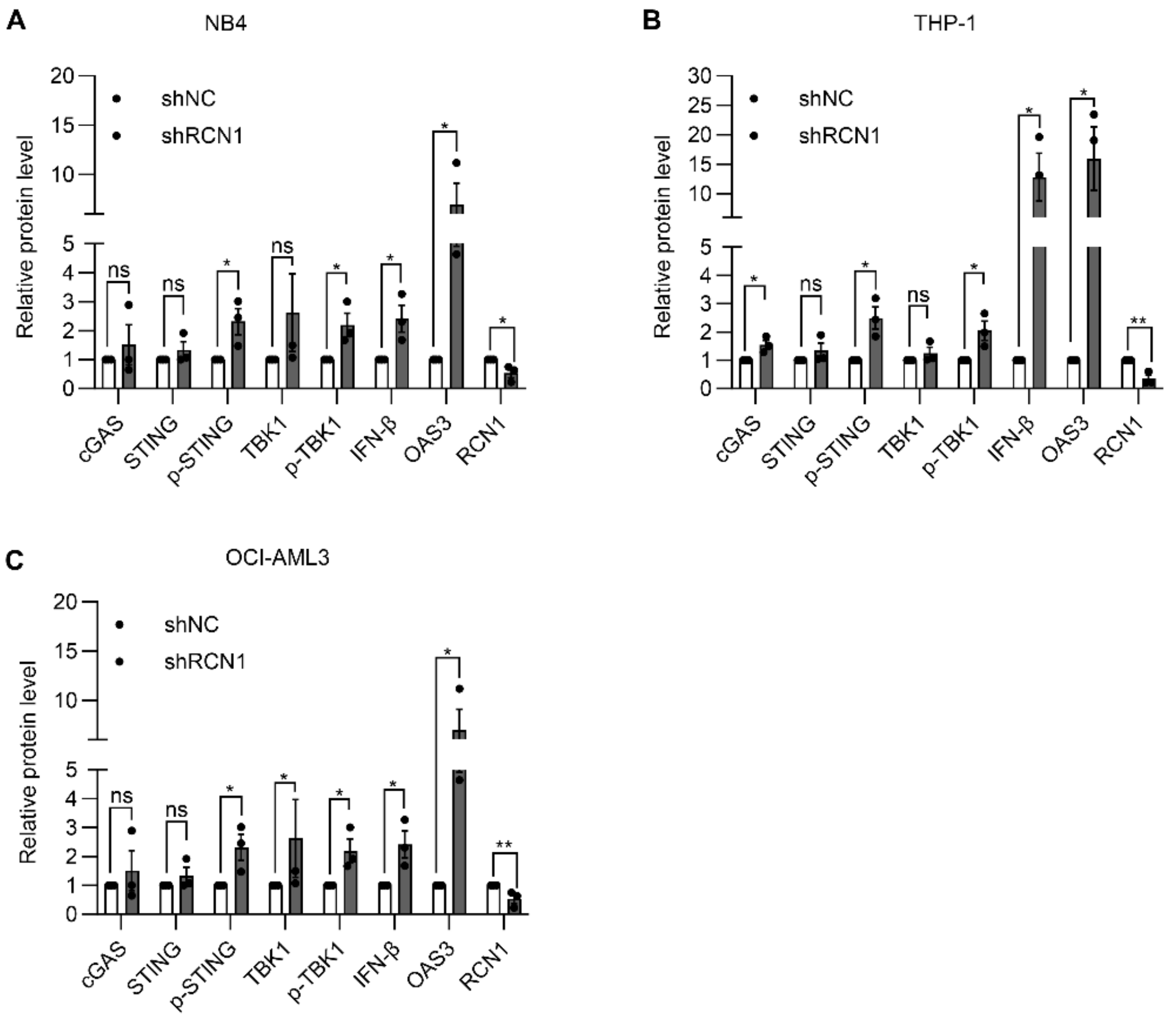

Supplementary Figure 1. Quantitative analysis of cGAS-STING signaling proteins following RCN1 knockdown in AML cells. (A-C) Densitometric quantification of RCN1, cGAS, STING, p-STING, TBK1, p-TBK1, IFN-β and OAS3 protein levels in NB4 (A), THP-1 (B) and OCI-AML3 (C) cells after RCN1 knockdown. Protein expression was normalized to β-actin. Results are shown as mean ± standard deviation. Statistical significance was assessed using a two-tailed unpaired *t*-test. ns, not significant; *P< 0.05, **P< 0.01.

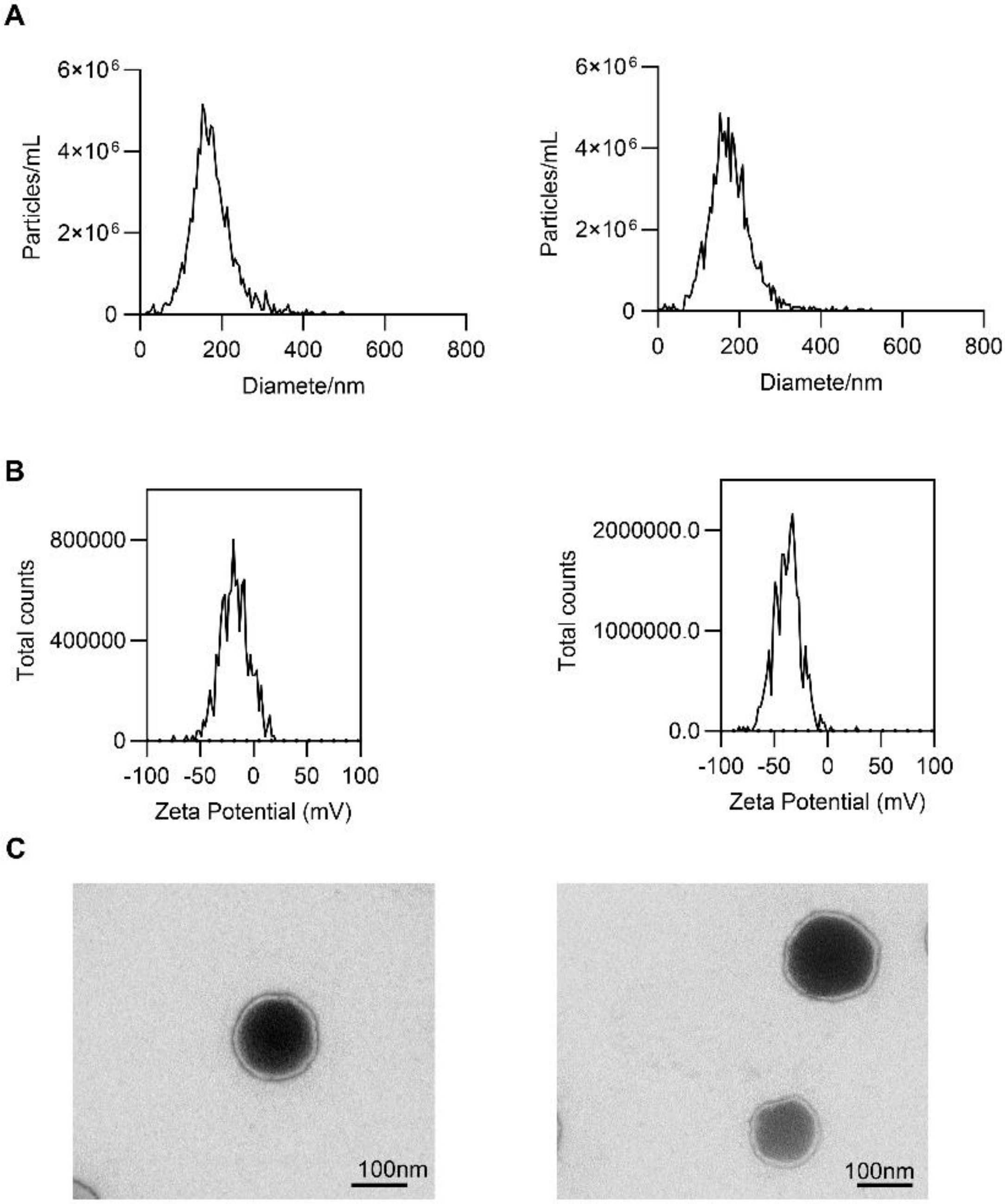

Supplementary Figure 2. Characterization of RBCEVs. (A) Particle size distribution profiles of two additional independent RBCEV batches determined by ZetaView analysis. (B) ZetaView analysis revealed the zeta potential of two additional independent RBCEV batches. (C) Representative TEM images showed the morphology of two additional independent RBCEV batches. The scale bar indicates 100 nm. TEM, Transmission electron microscopy.

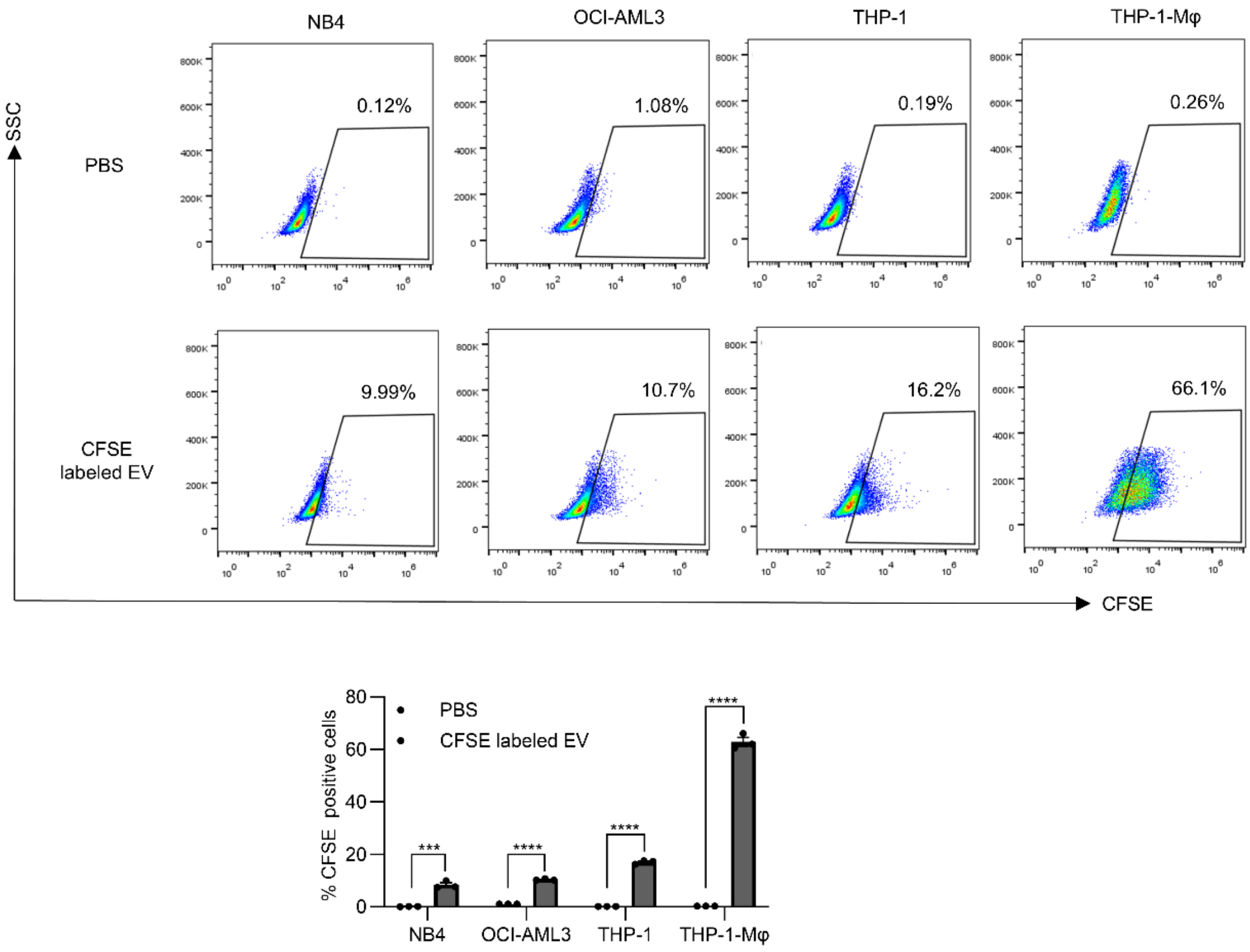

Supplementary Figure 3. Cellular uptake of RBCEVs in AML cells and THP-1-derived macrophages. NB4, OCI-AML3, THP-1 cells, and THP-1-Mφ were incubated with CFSE-labelled RBCEVs, and uptake was quantified by flow cytometry as the percentage of CFSE-positive cells. Statistical significance was determined using a two-tailed unpaired t-test. ***$P < 0.001$, ****$P < 0.0001$. CFSE, Carboxyfluorescein succinimidyl ester. THP-1-Mφ, THP-1-derived macrophages.

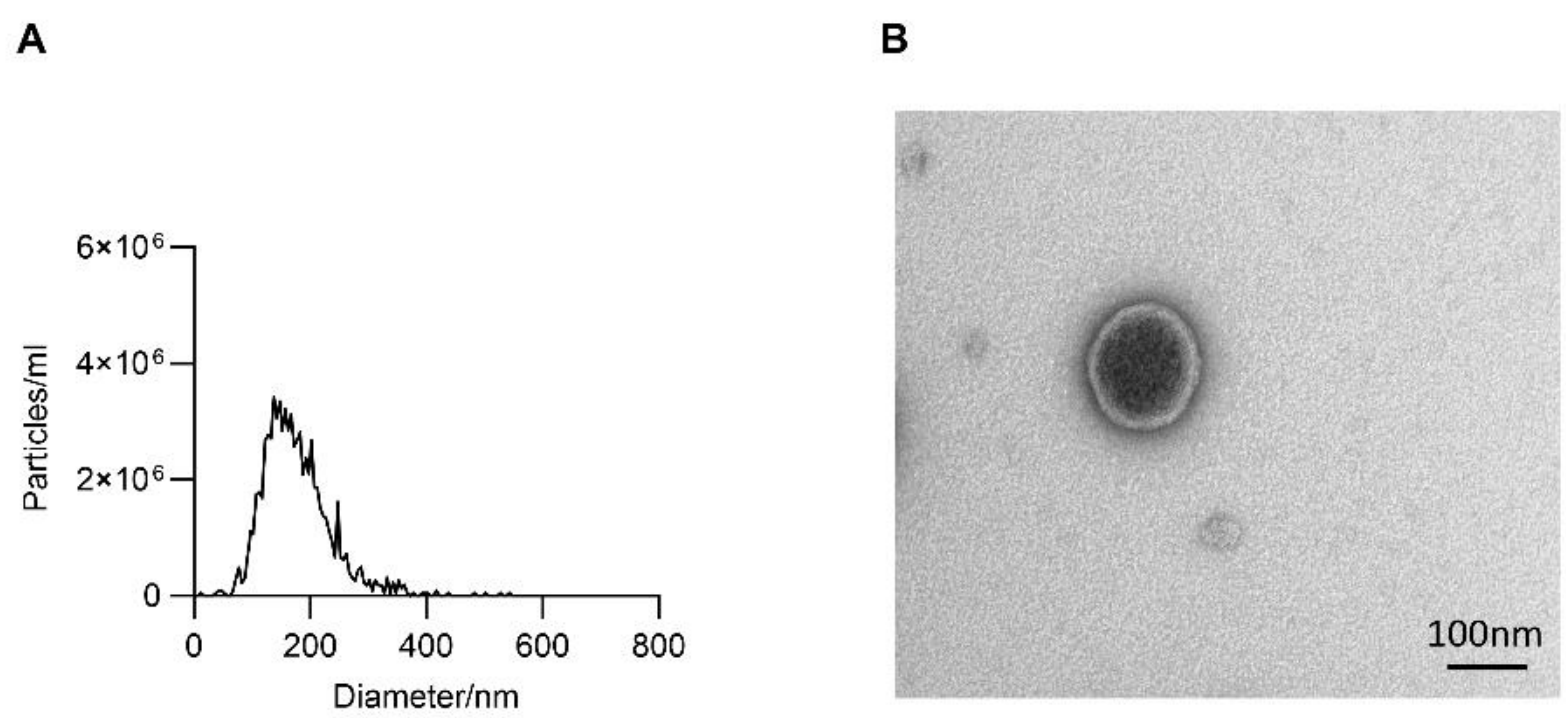

Supplementary Figure 4. Characterization of Cy5-siRNA loaded EV. (A) Particle size distribution profiles of Cy5-siRNA loaded EV determined by ZetaView analysis. (B)Transmission electron microscopy revealed that Cy5-siRNA loaded RBCEVs maintained a typical and intact vesicular morphology after siRNA loading. The scale bar indicates 100 nm.

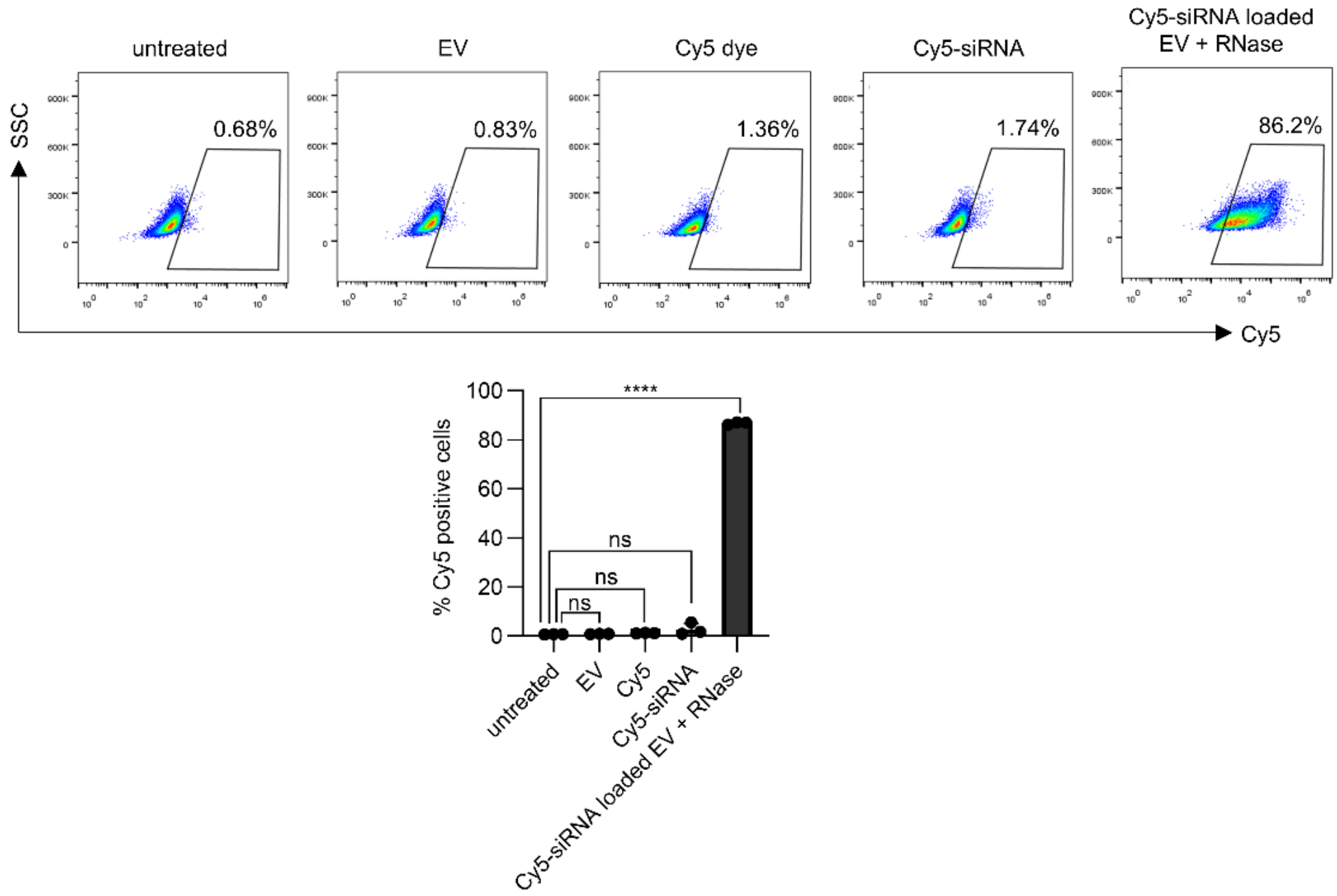

Supplementary Figure 5. Flow cytometric analysis of THP-1 cells treated with unloaded RBCEVs, free Cy5 dye, free Cy5-siRNA, or RNase-treated Cy5-siRNA loaded RBCEVs. Untreated THP-1 cells were used as a negative control. Cellular uptake was quantified as the percentage of Cy5-positive cells. Cy5 was conjugated to the siRNA cargo. Statistical significance was analyzed using one-way ANOVA followed by Tukey's multiple comparisons test. Data are presented as mean ± SD (*n* = 3). ns, not significant; ****$P < 0.0001$. Cy5, Cyanine 5.

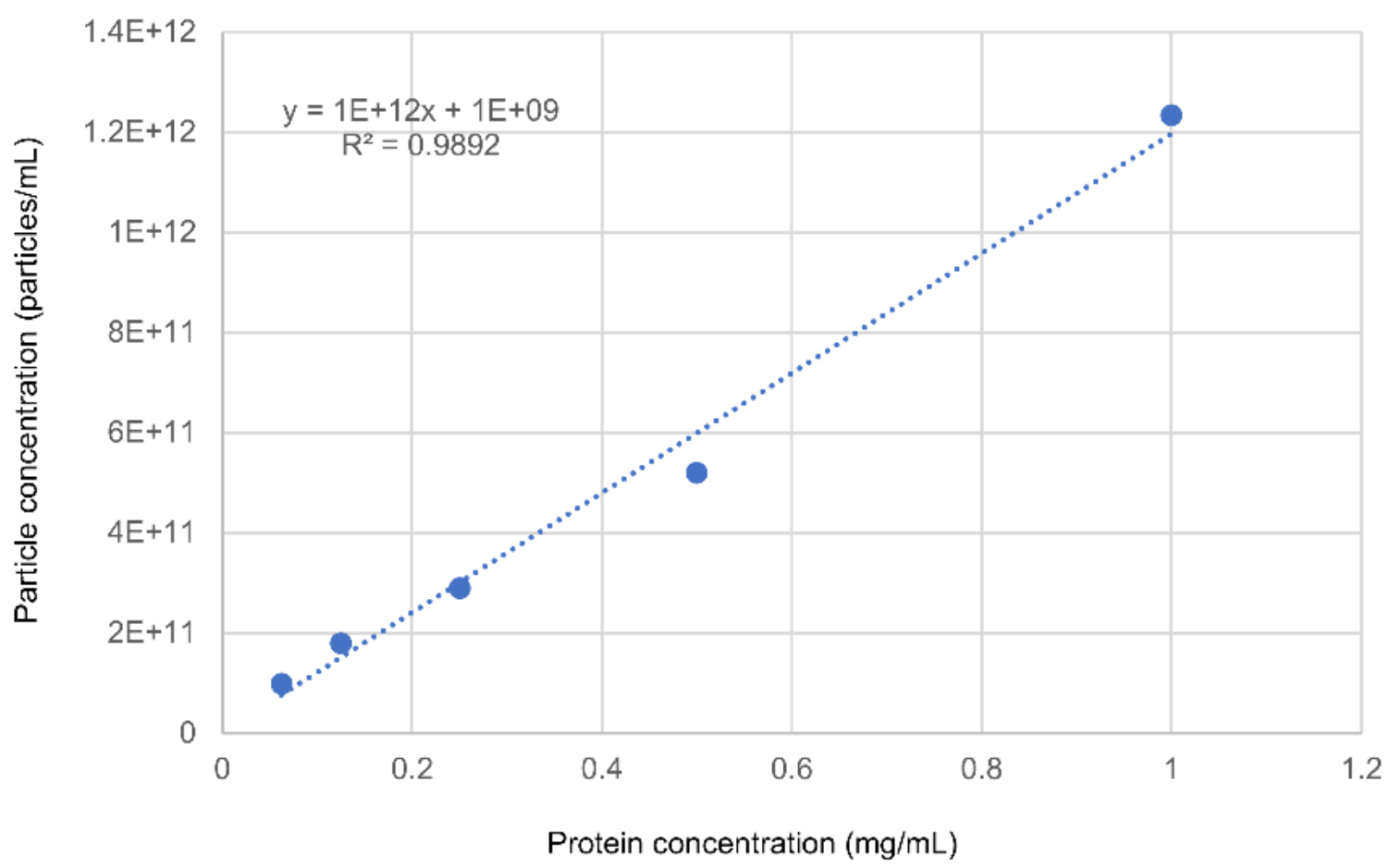

Supplementary Figure 6. Correlation between RBCEV protein concentration and particle number. Protein concentration of RBCEVs was determined by BCA assay, and particle concentration was measured using the ZetaView nanoparticle tracking analyzer. Linear regression analysis demonstrated a strong positive correlation between protein concentration and particle number ($R^2 = 0.9892$).

Supplementary Table 1. Sequences for gene knockdown

Name	Sequence (5'-3')
shNC	UUCUCCGAACGUGUCACGUTT
shRCN1	UCAUCUUUGUCUAACUUCCCG
shTFAM	GGCGGAGTGGCAGGTATATAA

Supplementary Table 2. List of primers

Primer name	Sequence (5'-3')
OAS1 Forward	CACCAAGCTCAAGAGCCTCA
OAS1 Reverse	TGTTTTCATGCTCCCTCGCT
IFIT1 Forward	GCCTTGCTGAAGTGTGGAGGAA
IFIT1 R1Reverse	ATCCAGGCGATAGGCAGAGATC
IFI27 Forward	CGTCCTCCATAGCAGCCAAGAT
IFI27 Reverse	ACCCAATGGAGCCCAGGATGAA
RSAD2 Forward	CCAGTGCAACTACAAATGCGGC
RSAD2 Reverse	CGGTCTTGAAGAAATGGCTCTCC
ISG15 Forward	CTCTGAGCATCCTGGTGAGGAA
ISG15 Reverse	AAGGTCAGCCAGAACAGGTCGT
TFAM Forward	GTGGTTTTCATCTGTCTTGGCAAG
TFAM Reverse	TTCCCTCCAACGCTGGGCAATT
COXI Forward	GCCCCAGATATAGCATTCCC
COXI Reverse	GTTCATCCTGTTCCTGCTCC
β2M Forward	TGCTGTCTCCATGTTTGATGTATCT
β2M Reverse	TCTCTGCTCCCCACCTCCAAGT

Supplementary Table 3. List of siRNA sequences

Name	Sense (5'-3')	Anti-sense (5'-3')
siNC	UUCUCCGAACGUGUCACGUT<br>T	AACGUGACACGUUCGGAGAA
siRCN<br>1	UCAUCUUUGUCUAACUUCCC<br>G	CGGGAAGUUAGACAAAGAUG<br>A
